# Matrix-assisted laser desorption ionization-time-of-flight mass spectrometry in veterinary medicine: Recent advances (2019–present)

**DOI:** 10.14202/vetworld.2022.2623-2657

**Published:** 2022-11-21

**Authors:** Jonathan E. Thompson

**Affiliations:** School of Veterinary Medicine, Texas Tech University, Amarillo, Texas 79106, United States

**Keywords:** biotyping, imaging, matrix-assisted laser desorption ionization-time-of-flight, microorganism identification, proteomics, veterinary diagnostics

## Abstract

Matrix-assisted laser desorption ionization-time-of-flight (MALDI-TOF) mass spectrometry (MS) has become a valuable laboratory tool for rapid diagnostics, research, and exploration in veterinary medicine. While instrument acquisition costs are high for the technology, cost per sample is very low, the method requires minimal sample preparation, and analysis is easily conducted by end-users requiring minimal training. Matrix-assisted laser desorption ionization-time-of-flight MS has found widespread application for the rapid identification of microorganisms, diagnosis of dermatophytes and parasites, protein/lipid profiling, molecular diagnostics, and the technique demonstrates significant promise for 2D chemical mapping of tissue sections collected postmortem. In this review, an overview of the MALDI-TOF technique will be reported and manuscripts outlining current uses of the technology for veterinary science since 2019 will be summarized. The article concludes by discussing gaps in knowledge and areas of future growth.

## Introduction

Matrix-assisted laser desorption ionization coupled to time-of-flight (MALDI-TOF) mass spectrometry (MS) has become a crucial diagnostic tool in the veterinary sciences. Despite the high cost of initial acquisition of the mass spectrometer (approx. $200–400k), the cost of running samples is very low (<$1/sample), making the technique attractive for high-throughput laboratories. The past decade has seen an explosion of uses of MALDI-TOF for microorganism identification based on the measurement of unique proteins specific to certain organisms. Through the creation of searchable mass spectral libraries, identification of microorganisms can occur with high confidence in a matter of minutes after cell culture steps are complete.

The applications for MALDI-TOF have grown substantially during the past decade, with applications in entomology, parasitology, microbiology, and proteomics consistently expanding. The technique has already been the topic of several crucial review articles, with many published before the COVID era (<2020) [1–7]. Two additional review articles have more recently appeared in the literature dealing specifically with use of MALDI-TOF for the identification of pathogenic microorganisms and virus identification [8, 9]. Nonetheless, the applications involving MALDI-TOF have continued to develop and expand since 2019. Therefore, this review aimed to focus on articles relevant to veterinary science that have appeared in peer-reviewed literature since 2019.

To discover recent literature, the Scopus online search engine was used, and 138 articles related to MALDI-TOF and veterinary medicine returned from 2019 to 2022 during the initial literature search. Note, the total reference count for this manuscript is above this figure since certain articles outside of the target date range have been cited to better present the discussion and alternate works have been uncovered during the literature search process which are relevant but did not make the initial list. Notwithstanding the exact reference count, the focus of this manuscript is clear; to provide veterinary professionals with an overview of MALDI-TOF technology and the state-of-the-science regarding MALDI-TOF as applied to veterinary diagnostics and research.

## Introduction to the MALDI-TOF Technique

Matrix-assisted laser desorption ionization-time-of-flight was reported initially by the research group of Franz Hillenkamp in 1987, with follow-up work by K. Tanaka of Shimadzu published in 1988 [10–12]. These early works established that when a light-absorbing component referred to as the “Matrix” was added to the sample, light from a high-peak-power ultraviolet (UV) Laser could be used for Desorbing and Ionizing a variety of high-molecular-weight biomolecules. The matrix, mixed with sample, essentially Assists the desorption/ionization process through physical mechanisms which are still not fully understood. These discoveries were revolutionary at the time because biomolecules up to 67 kDa were able to be desorbed into the gas phase and ionized as intact molecules! Before this work, volatilization of such large non-volatile molecules was unheard of and thought impossible. The fact the MALDI ionization is considered a “soft” ionization (no fragmenting molecules), is equally relevant since gas-phase molecules produced could be analyzed and molecular weights determined empirically to approx. 0.1% error (or better) through time-of-flight (TOF) MS.

**Figure-1:**
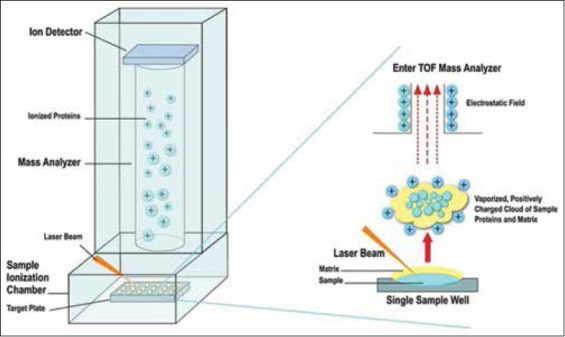
Schematic illustrating matrix-assisted laser desorption ionization time-of -flight (TOF) mass spectrometry. A pulsed ultraviolet laser (usually nitogen laser, 337 nm or Nd: YAG at 266 nm or 355 nm) focuses light onto a sample spot deposited and dried on a stainless steel target plate. The light absorbing matrix is mixed with the sample before deposition on the target. The target plate is placed within the sample ionization chamber held at high vacuum (<10^-6^ mbar). The figure on the right illustrates the desorption/ionization process which occurs after the laser pulse. Ions of the sample molecules are accelerated toward a long tube (approx. 1 m) called the drift tube or mass analyzer. Sample ions conduct a race from the tube entrance to the ion detector at the distal end of the tube, a race which is timed. The TOF or time required to traverse the tube can be correlated to the mass of the ion through an instrument calibration process. [Source: Image reproduced from https://doi.org/10.3390/jof5010004 under Creative Commons License].

In TOF, ionized molecules are collimated into a beam and accelerated to the same kinetic energy using conductive electrodes set at a high voltage. Figure-1 depicts the MALDI-TOF process. Kinetic energy (K.E.) is equal to:

K.E. = ½ mv^2^ (1)

Moreover, all ions accelerate to equal kinetic energy; ion velocities must be different because ion masses are not identical. More massive ions move at a slower velocity, while light ions have higher velocities. After acceleration, all ions are directed through a flight tube, usually, a large diameter stainless steel tube under high vacuum (<10^-7^ mbar) to prevent ions colliding with gas molecules. The ions traverse the tube and the time required to travel the tube is noted electronically. Through calibration, the flight time is correlated to molecular weight and a mass spectrum is obtained. Despite the benefits of MALDI ionization, it is quite inefficient. Estimates suggest that only a small fraction of analyte molecules are believed to be ionized by the MALDI process (roughly 1:10^4^–1:10^7^). TOF mass spectrometers have additional hardware options. One option is a reflectron, which decelerates ions and directs/reflects them to a second detector. The reflectron offers the benefit of increased mass resolution.

A second hardware option is a collision induced dissociation (CID). In CID, low pressure of an inert collision gas (often He) is purposefully leaked into the flight tube at roughly 10^-5^ mbar. Collisions between the gas and analyte cause fragmentation of analyte. Since a precursor ion can be isolated using an ion gate, this allows two-dimensional MS (so called MS/MS) to be carried out. The knowledge of precursor ion mass and fragment masses allows informed searching of online protein databases such as MASCOT (https://www.matrixscience.com), which frequently allows identification of proteins.

One important factor for MALDI measurements is the choice of matrix. Procedurally, a solution of the matrix is prepared to concentrations of ~5 mg/mL up to the point of solute saturation in a high-purity solvent such as water, methanol, or acetonitrile. Usually, a source of protons (H^+^) is added by inclusion of an easily evaporated acid such as 0.1% trifluoroacetic acid (TFA) or formic acid. The matrix solution is often mixed with sample solution on the stainless steel MALDI plate before allowing diffusional mixing and subsequent drying of the spot.

**Figure-2:**
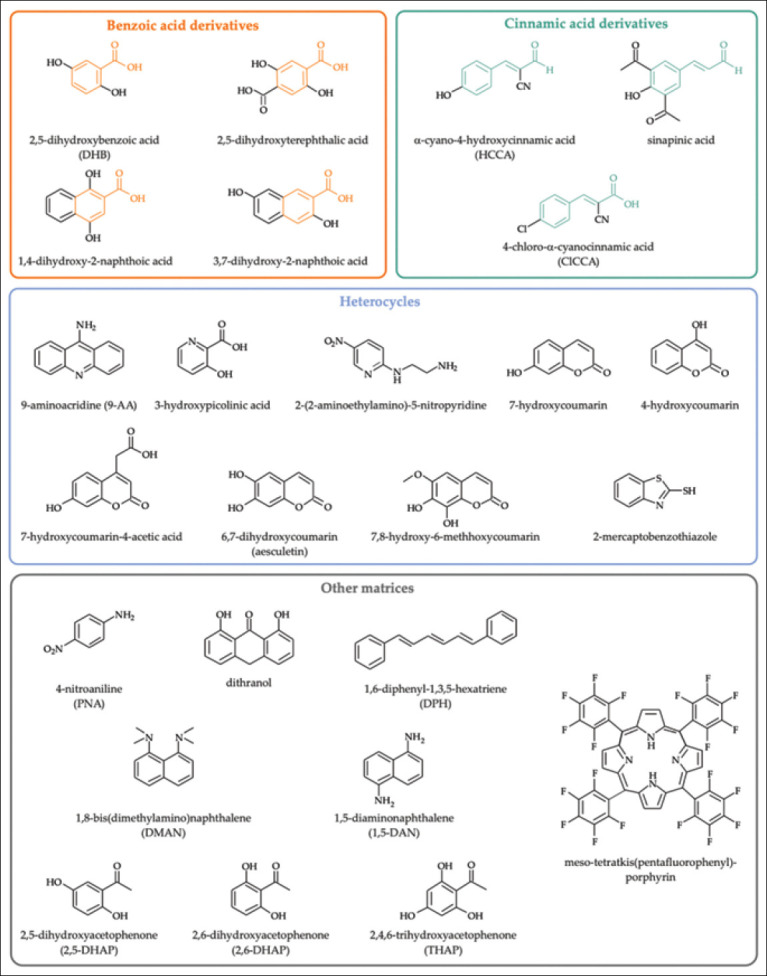
Common organic matrix materials for matrix-assisted laser desorption ionization time-of-flight mass spectrometry. Reproduced from Leopold *et al*. [13] under the Creative Commons Attribution License.

The composition of the matrix is an altogether different issue to consider. Fundamentally, the matrix material must satisfy five key criteria, including (1) strong light absorption in the UV range, (2) having adequate solubility in polar solvents and optimally being proton donating, (3) stable under storage and high vacuum conditions, (4) prevent analyte cluster formation from forming in the spectrometer source, and (5) form homogeneous crystals with biomolecules when drying/precipitating. Historically, three classes of matrix exist which satisfy the criteria: (1) Organic molecular matrix, (2) liquid crystals, and (3) inorganic materials such as metallic or graphitic nanoparticles. Of the classes, organic molecular matrices have demonstrated by far highest sensitivity for proteins and this class of matrix is almost universally used today. Organic molecular matrices are most popular of the classes, and tens of compounds have been studied for use as MALDI matrix material. Figure-2 illustrates chemical structures of some compounds which have been explored for use as a MALDI matrix [13]. Very common matrix materials include 2,5-dihydroxybenzoic acid, sinapinic acid, and α-cyano-4-hydroxycinnamic acid (often called CHCA or α-cyano). It should be noted that matrix is often matched to application. For instance, sinapinic acid, CHCA, and caffeic acid are most often used when ionizing large proteins due to improved ionization. Development of novel matrix materials and methods of application to the sample remain an important area of research.

Another vital factor to consider is sample preparation before MALDI analysis. Sample processing/preparation steps are typically application specific but in general, are guided by the principle that peptides and proteins present in the sample must be freed or extracted from the sample’s matrix completely. At simplest, this involves placing the sample into a solvent. Grinding, sonication, mixing/vortexing, or heating steps may follow. The exact composition of the solvent also frequently changes depending on the application. A very gentle solvent may be water with 0.1% formic acid or 0.1% TFA present, which would essentially simply dissolve the material present in the sample. Aqueous solvents may also be mixed with methanol, ethanol, or acetonitrile at various mixing ratios. For recalcitrant samples such as fungi, yeasts, certain Gram-positive bacteria, or insects, solvents are often supplemented with far higher concentrations of formic acid – up to 70%. These more aggressive solvents are required to adequately breakdown cellular material and tissues to and solubilize and extract proteins present. In addition, certain samples must be homogenized using laboratory devices such as the TissueLyser, using glass beads to help disrupt the material. Often, this step occurs within a homogenization buffer composed of a mix of 70% (v/v) formic acid and various volume mixing ratios of organic solvents in water.

If adequate sample preparation protocols are not developed and followed, MALDI mass spectra will not reflect the full molecular diversity possible, and library searches may fail to identify targets. For methods under development, trial and error may be necessary to develop a suitable sample digestion protocol. Literature can also be consulted to uncover protocols; other investigators have used successfully previously. It is crucial to understand the sample preparation step is vital to a successful implementation of MALDI-TOF and this should never be understated or underappreciated by end-users.

## Matrix-assisted Laser Desorption Ionization-time-of-flight for Microorganism Identification Applied to Veterinary Science

The literature review conducted suggests the area of the highest current application of MALDI-TOF in veterinary sciences is microorganism identification. The premise behind the science is that each microorganism expresses a unique set of proteins between 2000 and 20,000 Da molecular weight, and peaks present in a MALDI mass spectrum can be used to identify organisms through library searching aided by a computer. Manufacturers of MALDI-TOF hardware have developed spectral libraries over the past decade, which have proven quite useful in identifying microorganisms with high confidence. At present, three major MALDI-TOF spectral libraries have emerged as the most used. The “best” library is a subject of intense debate among investigators, with opinions often influenced by specific applications or organisms of interest. All libraries contain a very large number of organisms (hundreds – thousands). As it is beyond the scope of this manuscript to define “best” library, I will simply direct the reader to publicly available documents listing the organisms covered for each of the three libraries commercially available. Documents listing the organisms present within the commercial libraries (Bruker - Billerica, MA, USA, Shimadzu - Columbia, MD, USA, Biomerieux - Marcy-l’Etoile, France) are available in the supplemental information for this manuscript. In addition to the commercial libraries, end-users are frequently able to add high-quality MALDI-TOF spectra to their own local libraries to allow expansion for the identification of a particular species of interest. On occasion, end-users or government agencies develop and disseminate spectral libraries for public use [14–16]. Application of MALDI-TOF to microorganism identification is far-reaching and the remainder of this text section, and Table-1 outlines recent developments in the field [17–116].

**Table-1:** List of recent application of MALDI-TOF for microorganism identification.

Species identified	Notes	References
*Tenacibaculum finnmarkense*	Ulcerative lesions in Atlantic salmon	[17]
*Iodobacter limnosediminis*	Causes skin lesions in freshwater fish	[18]
*Streptococcus iniae*	Outbreak investigation	[19]
*Vibrio*	23 reference spectra published for aquaculture	[20]
*Tenacibaculum maritimum*	Causes tenacibaculosis in marine fish	[21]
*Streptococcus agalactiae, Streptococcus iniae, Aeromonas hydrophila, Aeromonas veronii,* and *Edwardsiella tarda*	Spectral library of 75 isolates related to aquaculture	[22]
Various	Mastitis diagnosis improved by MALDI	[23]
*Staphylococcus aureus, Escherichia coli, Klebsiella pneumoniae, Staphylococcus epidermidis, Streptococcus uberis, Bacillus clausii,* and *Corynebacterium amycolatum*	Causes of mastitis in dromedary camels	[24]
Staphylococci, *Corynebacterium* spp., and *Bacillus* spp.	How teat microbiome is affected by disinfection with lactic acid and chlorhexidine	[25]
*Staphylococcus* spp., *Micrococcus luteus*, *Corynebacterium* spp., *Bacillus* spp., *Escherichia coli*, *Enterobacter cloacae*, *Aerococcus viridans*, *Morganella morganii*, and *Turicella otitidis*	Causes of caprine mastitis	[35]
*Mycoplasma bovis*	Cause of respiratory disease, arthritis, and mastitis in the beef and dairy industry	[26]
*Streptococcus uberis*	Prediction of positive treatment outcome	[27]
*Streptococcus uberis*	Mastitis in Brazilian dairy cattle	[28]
*Prototheca bovis, Prototheca blaschkeae*, and *Pichia ciferrii*	Improved diagnosis of bovine mastitis	[29]
Staphylococci	Bovine mastitis and antibiotic resistance	[30]
Staphylococci	Mastitis in a donkey	[31]
Non-aureus *Staphylococcus*	Mastitis in water buffalo	[32]
*Streptococcus, Enterococcus, Lactococcus*, and *Aerococcus*	Bovine mastitis in Brazil	[33]
*Staphylococcus aureus*	Bovine mastitis/machine learning for prediction of antibiotic resistance	[34]
Staphylococci	Mastitis in Brazilian dairy goats	[35]
*Staphylococcus chromogenes, Staphylococcus* spp., *Aerococcus* spp., *Lactococcus* spp., *Bacillus* spp., and *Corynebacterium* spp.	Bovine mastitis; prevalence of various causative organisms	[36]
*Staphylococcus aureus*	Mastitis in cows, sheep, goats, and camels in Saudi Arabia	[37]
Over 50 organisms	Diagnosis in subclinical stage of mastitis in ewes	[38]
*Candida albicans*	Bovine mastitis	[116]
*Staphylococcus sciuri, Staphylococcus chromogenes, Staphylococcus haemolyticus, Staphylococcus xylosus, Staphylococcus hyicus,* and *Staphylococcus warneri*	Mastitis in cattle and buffaloes in Egypt	[39]
Non-aureus Staphylococci	Bovine mastitis samples	[40]
Non-aureus Staphylococci	Bovine mastitis samples	[41]
*Staphylococcus aureus* and environmental streptococci	Bovine mastitis, milk yield	[42]
*Streptococcus agalactiae, Staphylococcus aureus, Staphylococcus simulans, Staphylococcus delphini, Staphylococcus rostri, Staphylococcus chromogenes, Staphylococcus hyicus, Staphylococcus haemolyticus,* and *Staphylococcus warneri*	Mastitis in *Camelus dromedarius* herds in Kenya	[43]
*Nocardia farcinica*	Bovine mastitis, first documented case from organism	[44]
*Staphylococcus argenteus*	Mastitis	[45]
*Serratia marcescens*	Mastitis outbreak on a farm, contaminated teat dip source	[46]
Non-aureus *Staphylococcus*	Comparison of MALDI-TOF with 16S sequencing	[47]
*Erysipelothrix rhusiopathiae*	Case study/investigation	[48]
*Staphylococcus* spp.	Human/pet transmission and antibiotic resistance	[49]
Non-TB mycobacteria	>75% of isolates identified, but database improvements still needed	[50]
*Trueperella pyogenes*	Uterine bacteria in cows, reproductive effects	[51]
*Staphylococcus aureus*	Antibiotic resistance in swine	[52]
*Comamonas kerstersii*	Case study of mortality in young goat	[53]
*Klebsiella variicola*	Case study, equine respiratory distress	[54]
*Enterococcus*	Investigation of pet food contamination	[55]
*Campylobacter*	Organism surveillance	[56]
*Escherichia coli* and *Enterococcus*	>80% of samples antimicrobial resistant from anal swab samples, various species	[57]
Various organisms	Survey of organisms, antimicrobial resistance in wildlife from Gabon	[58]
*Salmonella*	1.96%–3.2% of turtles tested positive for *Salmonella*	[59]
Various organisms	New spectral library for equine bacterial infections	[60]
*Lactococcus garvieae*	Investigation of strain origins obtained from nasal swab of dog	[61]
*Treponema* spp.	Identification of bacteria causing digital dermatitis	[62]
*Aerococcus viridans*	Causes of abortion in swine	[63]
*Staphylococcus pseudintermedius*	Racoon dogs in Shandong Province China, zoonosis	[64]
*Bartonella refiksaydamii*	Novel species of *Bartonella* from lesser shrew in Turkey	[65]
*Klebsiella pneumoniae*	Colistin resistance profile of *Klebsiella*	[66]
*Staphylococcus* spp.	Prevalence in broiler chickens and b-lactam resistance	[67]
*Staphylococcus*	*Staphylococcus* in dogs and antimicrobial resistance	[68]
*Escherichia coli*	Feces from Brazilian dogs and cephalosporin resistance	[69]
Various organisms	Bacteria in air samples at veterinary hospital	[70]
*Escherichia coli,* various organisms	Canine urinary tract infections	[71]
*Pasteurellaceae*	Investigation of multi-species mortality event in Florida	[72]
*Moraxella bovoculi*	Bovine pink eye and RTX toxin	[73, 98]
Staphylococci and mammaliicocci	Species confirmation from nasal swabs from cattle, calves, goats, sheep, llamas, and alpacas – also antimicrobial resistance	[74]
*Escherichia coli*	Samples from feces of raccoons in Europe, antimicrobial resistance profiles	[75]
*Clostridioides difficile*	Fecal samples collected from trash cans in public gardens	[76]
*Escherichia coli*	Prevalence in chickens in Tanzania and antibiotic resistance	[77]
*Staphylococcus pseudintermedius*	Case study of mortality in young bitch	[78]
*Brucella canis* and *Brucella suis*	Trained software to identify *Brucella canis* from a closely related *Brucella suis*	[79]
Various equine pathogens	Screening of equine health, spectral library development	[60, 80]
*Vagococcus lutrae* and *Vagococcus fluvialis*	Brazilian swine	[81]
Various organisms	Studied puppy meconium microbiota as function of type of delivery	[82]
*Staphylococcus*	From various species over 10 years and antibiotic resistance	[83]
Mycoplasmas	MALDI for screening of poultry flocks	^[84]^
*Enterococcus*	In Japanese race horses and multidrug resistance profiles	[85]
*Enterobacteria*	Fecal samples from turkeys, widespread antibiotic resistance found	[86]
*Trueperella pyogenes*	Samples include vaginal discharge of an okapi (*Okapia johnstoni*) and the kidney of a female royal python	[87]
*Escherichia coli*, *Salmonella*, and *Acinetobacter*	Study of backyard poultry flocks in the United States	[88]
*Clostridium botulinum*	Identified peptide biomarkers to identify botulism in cattle	[89]
Various organisms	Studied how aseptic skin preparation techniques affected bacterial counts prior to surgery	[90]
*Staphylococcus cohnii, Bordetella bronchiseptica, Bordetella parapertussis, Corynebacterium glucuronolyticum, Pelistega suis*, and *Rodentibacter rarus*	Screening of deceased street rats in France	[91]
*Staphylococcus pseudintermedius*	Canine pyoderma, antibiotic resistance	[92]
*Streptococcus suis*	Identified/confirmed *Streptococcus suis* in samples from swine	[93]
*Streptococcus suis*	Isolated from swine, tested for antimicrobial resistance, large fraction resistant to tetracycline	[95]
*Klebsiella pneumoniae*	Fecal samples from dogs, dogs can be important carriers of extended-spectrum beta-lactamase producing *Klebsiella*	[96]
*Mycoplasma bovis*	Samples obtained from cattle bronchoalveolar lavage fluid	[97]
*Moraxella bovoculi*	Screening samples for Bovine keratoconjunctivitis	[98]
*Staphylococcus pseudintermedius*	Methicillin-resistant *Staphylococcus pseudintermedius* in dogs/cats	[99]
Various organisms (>300)	Range of aerobic bacteria in skin lesions of reptiles	[100]
*Arcanobacterium phocae*	Strains isolated from cases of mink dermatitis	[101]
*Brucella* spp.	Survey of bovine and buffalo dairy herds in the Nile delta region of Egypt	[102]
*Listeria monocytogenes*	Case study of lambs/ewes in Kosovo	[103]
*Bibersteinia trehalosi*	Investigation of illness in 15 calves	[104]
*Staphylococcus* spp.	Prevalence in clinically healthy goats (nasal swabs)	[105]
*Staphylococcus*	Prevalence in Algerian sheep	[106]
Various	Testing use of human diagnostic kit for sepsis in dogs/cats	[107]
Various	Optimizing cell culture conditions before MALDI	[108]
*Bergeyella zoohelcum*	Survey of abundance in mouths of therapy dogs	[109]
*Staphylococcus pseudintermedius*	Survey of skin of cats/dogs	[110]
*Corynebacterium ulcerans*	Develop method for reliable speciation between members of the *Corynebacterium diphtheriae* group	[111]
*Mycoplasma bovis*	Developed rapid culture based assay	[112]
*Trueperella abortisuis*	Initial detection of organism in companion animals	[113]
*Mycoplasma*	Developed MALDI method for rapid screening of avian mycoplasma	[114]
Various fungi	Validation of a free online spectral database for veterinary fungi, 90% matching success	[115]

MALDI-TOF=Matrix-assisted laser desorption ionization-time of flight

The use of MALDI-TOF to rapidly identify infectious outbreaks on aquaculture farms is an emerging application of the technique. Spilsberg *et al*. [17] used MALDI-TOF to investigate bacterial origins of ulcerative lesions in Atlantic salmon. After training a reference library with known samples, the authors identified *Tenacibaculum finnmarkense* and *Tenacibaculum* *ulcerans* in 13/15 and 7/15 outbreaks of ulcerative lesions on the western coast of Norway. Korkea-Aho *et al*. [18] used MALDI-TOF to identify *Iodobacter limnosediminis* present in samples of freshwater fish in Finland. This species is associated with skin ulcers and saprolegniosis. Matrix-assisted laser desorption ionization-time-of-flight can also be used in an investigative manner. Young *et al*. [19] used MALDI-TOF to identify *Streptococcus iniae* during the March 2016 outbreak in aquaculture off the coast of Australia. The work highlights MALDI-TOF’s ability to rapidly identify causative agents in outbreaks. Vibriosis (infection of *Vibrio* bacteria) is a major cause of mass mortality events and corresponding economic losses on aquaculture farms. Mougin *et al*. [20] have developed a new in-house database named Luvibase, composed of 23 references MALDI-TOF mass spectra profiles obtained from *Vibrio* collection strains for monitoring and rapid diagnosis of *Vibrio*. The authors publish reference spectra for the most common *Vibrio* species affecting aquaculture, thereby providing a valuable resource for biotyping. Bridel *et al*. [21] have also considered this emerging problem in commercial aquaculture – the dramatic increase in outbreaks of infectious disease on commercial farms. One specific concern is tenacibaculosis, a serious bacterial infection which can affect many commercial marine fish species, leading to considerable economic losses. *Tenacibaculum maritimum* is the best characterized organism, leading to this condition. In this paper, the authors sequenced and compared 25 genomes for isolates obtained from a variety of geographical locations. The authors used the genomic data to even predict m/z values for detected mass peaks that could be assigned to proteins. The authors could even detect shifts in m/z values for substitutions of specific amino acids in these proteins. The manuscript represents an impressive tour de force of what is possible by combining genomic data with MALDI-TOF biotyping. Piamsomboon *et al*. [22] also commented on detection of fish pathogens by MALDI-TOF. In their work, *Streptococcus agalactiae*, *S. iniae*, *Aeromonas hydrophila*, *Aeromonas veronii*, and *Edwardsiella tarda* isolates were obtained from diseased fish. Then, peptides and proteins were extracted from the bacteria and used to create library reference spectra for identification. A test set of 75 bacterial isolates yielded 100% correct matching when the updated database was used. The updated spectral library published in his work is a useful resource for the identification of these organisms by MALDI-TOF.

Another major veterinary focus area involving MALDI-TOF measurements is in the diagnosis of mastitis in milk-producing animals. Astrup *et al*. [23] determined that MALDI-TOF is a powerful tool for the diagnosis of clinical bovine mastitis. Using the MALDI analysis as a gold standard, the authors determined that out of 492 test cases, only 90 diagnoses made in veterinary clinics (18%) were correct. These results are highly significant since misdiagnosis may lead to the choice of wrong treatment regimen and hamper the prudent use of antibiotics. Ranjan *et al*. [24] used MALDI-TOF to discern that *Staphylococcus aureus*, *Escherichia coli*, *Klebsiella pneumoniae*, *Staphylococcus epidermidis*, *Streptococcus uberis*, *Bacillus clausii*, and *Corynebacterium amycolatum* were major players causing mastitis in dromedary camels in India. For the 1^st^ time, the study established the major species responsible for mastitis and quantified the economic impact of the condition on the region. In an interesting article, Schwenker *et al*. [25] utilized the microorganism identification feature of MALDI-TOF to note the microbiome composition on teats before and after disinfection with lactic acid and chlorhexidine. Such a study has broad implication for mastitis in dairy herds, which causes discomfort for animals in addition to economic losses stemming from decreased milk yield, treatment regimens, and in some cases premature culling. The authors learned that the abundance of coagulase-negative staphylococci (abbrev. CNS) decreased substantially for both the lactic acid and chlorhexidine treatments (64.3%–51% and 58.6%–39.6%, respectively). On the other hand, the percentage of samples indicating positive for *Corynebacterium* spp. and *Bacillus* spp. was observed to remain similar or even marginally increase after treatment with lactic acid or chlorhexidine. The ability of MALDI-TOF to offer high throughput of samples at a relatively low cost was instrumental to this study’s success. This work highlights MALDI-TOF’s contribution to the field of veterinary microbiology. In another work, a team from Brazil collected 714 caprine milk samples and used MALDI-TOF to characterize bacterial isolates present within the milk to characterize major pathogens causing mastitis [117]. The authors identified over 200 pathogens present, with major pathogens being *Staphylococcus* spp., *Micrococcus luteus*, *Corynebacterium* spp., *Bacillus* spp., *E. coli*, *Enterobacter cloacae*, *Aerococcus viridans*, *Morganella morganii*, and *Turicella otitidis*. The team also investigated the prevalence of antibiotic resistance genes through sequencing analysis to find the following frequencies observed: 64.60% *bla*Z, 2.25% *mec*A, 22.47% *tet*(L), 16.85% *tet*(M), 6.41% *tet*-38, 37.07% *nor*A, 14.04% *nor*B, 4.49% *nor*C, 7.30% *msr*A, 8.42% *van*A, and 0.56% *van*B. McDaniel and Derscheid [26] investigated MALDI-TOF for rapid and conclusive screening for *Mycoplasma bovis* – a crucial pathogen causing respiratory disease, arthritis, and mastitis in the beef and dairy industry. These authors report developing improved screening mechanisms is crucial as the traditional cumbersome assays require many weeks to detect the slow-growing organisms. The authors determined that MALDI-TOF worked well for the identification of *M. bovis* strains. Every *M. bovis* isolate was correctly identified as *M. bovis* by the MALDI-TOF software, and none of the field isolates were misidentified as one of the other mycoplasmas present in the library, nor were the other mycoplasmas misidentified as *M. bovis*. Maciel-Guerra *et al*. [27] used MALDI-TOF as a diagnostic tool for the prediction of positive treatment outcome for *S. uberis* infections in dairy herds. As influenced by the earlier work of Ebrahimi *et al*. [118], this fascinating work uses MALDI-TOF data coupled with machine learning to identify patterns in datasets to improve diagnosis. The basic goal was to identify diagnostic criteria to differentiate between cases which were responsive versus unresponsive (classes) to treatment regimens. A total of 90 cows were considered, and bacterial isolates from each were used to construct the working model. Matrix-assisted laser desorption ionization-time-of-flight peak lists with paired mass/charge (m/z) ratios and corresponding intensity values were extracted from the raw spectra for both classes. Welch’s t-test was used to detect statistically relevant differences in peak intensities between responsive and unresponsive classes. A variety of classification methods were investigated to achieve optimal performance for dichotomous decision making based on data. The authors also investigated and reported on the proteins found to correspond to the discriminatory signals in the MALDI spectrum. The work is a significant step forward as the diagnostic scheme allowed an accuracy of 92.2%, with a Cohen’s kappa score of 84.1% for the prediction of responsive versus unresponsive treatment outcomes. This remarkable result will allow management staff to make informed decisions regarding animal welfare and pursue the most appropriate treatment regimens. Martins *et al*. [28] also considered *S. uberis*, an organism which causes up to 17% of clinical mastitis cases in dairy cattle. In their work, they isolated colonies from dairy herds in the southeast of Brazil. Matrix-assisted laser desorption ionization-time-of-flight was used to confirm the identity of specifically *S. uberis*. Then, these confirmed isolates were treated with eight antimicrobials commonly used for clinical mastitis. Antibiotic resistance was assayed, with highest frequencies of resistance observed for erythromycin (80.7% resistant, R), tetracycline (R = 59%), and penicillin (R = 57.8%). Only 10.8% of *S. uberis* isolates were resistant against ceftiofur, and only 1.2% of *S. uberis* isolates were resistant to enrofloxacin. A separate Brazilian team (Fidelis *et al*. [29]) also used MALDI-TOF to improve diagnosis of bovine mastitis. In their work, they focused on identifying *Prototheca bovis*, *Prototheca blaschkeae*, and *Pichia ciferrii*. The authors claim *Prototheca* is an emerging cause of mastitis that is non-responsive to treatment with conventional antimicrobial agents. The authors acquired MALDI-TOF spectra for these species which they isolated and added the spectral features to the Bruker spectral library to be used for later identification. One key feature of this report is the authors describe an extensive sample preparation protocol to acquire the proteins needed for the MALDI-TOF matching. Preparing samples and extracting protein content are indeed one of the most critical aspects of biotyping by MALDI-TOF, and it is refreshing to see these authors consider this topic extensively in their work. Rowe *et al*. [119] also used MALDI-TOF to explore mastitis infections in dairy cattle. Nearly half of the 1594 cows sampled from 56 herds had at least one infected quarter. Matrix-assisted laser desorption ionization-time-of-flight was used for the identification of the organisms responsible for infection. The authors report *Staphylococcus chromogenes* infected 19.3% of cows with other common causes, including *Staphylococcus* spp. (6.8%), *Aerococcus* spp. (6.0%), *Lactococcus* spp. (5.3%), *Bacillus* spp. (5.5%), and *Corynebacterium* spp. (3.6%). In another bovine mastitis-related article, Fergestad *et al*. [30] used MALDI-TOF to identify species of staphylococci recovered from milk. The authors expanded the work by studying methicillin resistance in the recovered bacteria. The work of Podico *et al*. [31] demonstrates how MALDI-TOF can be a valuable tool in the diagnostic laboratory. This work chronicles how an 8-year-old donkey presented with anorexia, depression, and a painful right udder. Cytology of secretions from the affected area revealed many neutrophils and a diagnosis of mastitis was made. Matrix-assisted laser desorption ionization-time-of-flight analysis identified a bacterium of *Streptococcus* genus but failed to identify the species. Follow-up genome analysis revealed that the authors had identified a novel *Streptococcus* species. Singha *et al*. [32] attacked the issue of mastitis in water buffalo through MALDI-TOF biotyping. While clinical mastitis can often be diagnosed by observable changes in the milk quality, udder, and condition of animals, subclinical mastitis remains undetectable in most cases due to lack of obvious clinical signs. The authors sampled milk from 76 lactating buffalo at 16 buffalo farms in Bangladesh, cultured the milk, and recovered isolates. The authors indicate that non-aureus *Staphylococcus* was the most prevalent bacteria, presenting in approx. 25% of samples recovered. Oliveira *et al*. [33] used MALDI-TOF measurements to identify 380 bacteria isolates from cases of bovine mastitis in Brazil. This survey identified various *Streptococcus*, *Enterococcus*, *Lactococcus*, and *Aerococcus* species as major players infecting animals. Esener *et al*. [34] embraced the broad power of MALDI-TOF by acquired spectra for 82 *S. aureus* isolates collected from 67 cows diagnosed with mastitis. The authors correlated key spectral features with phenotypic demonstration of antibiotic resistance through 10 different supervised learning techniques (machine learning). Applying supervised learning to MALDI spectra is a very promising area of research for the future, with many new and exciting applications possible. This work demonstrates this well, as the authors were able to obtain a Cohen’s kappa of >95% for diagnosis. In the work of Bezerra *et al*. [35], half-udder milk samples were collected at the early (50 days), intermediate (100 days), and late stages of lactation (150 days) from dairy goats in Brazil. Matrix-assisted laser desorption ionization-time-of-flight was used for the identification of isolates causing mastitis in the goats. The authors found that staphylococci species accounted for the great majority of the isolates collected in the study (96.1%). Furthermore, intramammary infections significantly reduced fat and total solids in goat milk but raised somatic cell count and total bacteria count. The work of Rowe *et al*. [36] demonstrates the power of MALDI-TOF for understanding bovine mastitis. Similar to others, in this work, bacterial isolates collected from milk samples from 80 herds in 10 states were identified using a MALDI-TOF mass spectrometer. The work was comprehensive in scope, reporting a prevalence of over 20 species associated with mastitis. The unique nature of MALDI-TOF has truly empowered this study to be completed as it allows rapid, low-cost analysis with definitive results for biotyping of numerous species. Alharbi *et al*. [37] studied mastitis in cows, sheep, goats, and camels in their native Saudi Arabia using MALDI-TOF. Analysis of 400 milk samples revealed that *S. aureus* was a major trigger for mastitis in Saudi Arabia. Matrix-assisted laser desorption ionization spectra revealed peaks at 2636 Da, 3009 Da, 4590 Da, 4863 Da, and 4938 Da; the authors linked to methicillin-resistant *S. aureus* and methicillin-sensitive *S. aureus* (MSSA), respectively. The paper demonstrates not only MALDI-TOF’s ability to identify and type bacteria but also identify specific peaks in the mass spectrum which serve as biomarkers for methicillin-resistant or methicillin-sensitive strains. Knuth *et al*. [38] focused on the critical subclinical stage of mastitis in ewes. At this stage, infection is difficult to diagnose, yet intervention is crucial to prevent the further advance of the condition within the animal and herd. To improve subclinical diagnosis, the authors performed a physical examination of udder and teat traits while also collecting data on somatic cell counts and identify isolates of bacteria collected from milk by MALDI-TOF analysis. The authors identified more than 50 organisms, with *Bacillus licheniformis*, *Micrococcus flavus*, *Bacillus amyloliquefaciens*, and *S. epidermidis* being among the most common. In another work, El-Ashker *et al*. [39] utilized MALDI-TOF to identify bacteria present in subclinical cases of mastitis in cows and buffaloes in Egypt. For MALDI, the authors used the direct colony extraction technique in which material from a single colony was spotted twice on the same target, followed by adding 0.8 μL of CHCA matrix solution onto each sample. Matrix-assisted laser desorption ionization biotyping identified six different CNS from cattle and buffaloes (*Staphylococcus sciuri*, *S. chromogenes*, *Staphylococcus haemolyticus*, *Staphylococcus xylosus*, *Staphylococcus hyicus*, *Staphylococcus warneri*, and one unidentified species). Wuytack *et al*. [40] used MALDI-TOF for species-level identification of non-aureus staphylococci collected from bovine mastitis samples. The authors expanded the spectral identification library to include species of relevance to bovine clinical mastitis, including *Staphylococcus jettensis*, *Staphylococcus lentus*, *Staphylococcus rostri*, *Staphylococcus saprophyticus*, *S. chromogenes*, *S. epidermidis*, *Staphylococcus equorum*, *Staphylococcus fleurettii*, *S. haemolyticus*, *S. hyicus*, and *Staphylococcus simulans*. Alnakip *et al*. [41] utilized MALDI-TOF to uncover protein fingerprints that can be used to discriminate between the most prevalent major (*S. agalactiae*, *S. dysgalactiae*, and *S. uberis*) and minor (*Streptococcus canis*, *Streptococcus parauberis*, *Streptococcus salivarius*, *Streptococcus equinus*, and *Streptococcus gallolyticus*) streptococci involved in bovine mastitis. Matrix-assisted laser desorption ionization-time-of-flight profiling uncovered that *Streptococcus* spp. exhibits three genus-specific biomarkers peaks in the MS spectrum with m/z values of 2112, 4452, and 5955 Da. Gonçalves *et al*. [42] have reported on the effect of mastitis pathogens on dairy milk yield and composition. The authors used MALDI-TOF for bacterial identification after culturing. The ability to type pathogens allowed the authors to uncover a link between pathogen identity and milk yield. Yield decrease (0.8–1.3 kg/quarter) varied according to the infectious pathogen and was higher when associated with major pathogens such as *S. aureus* and environmental streptococci compared with healthy quarters. The authors also observed decreased milk fat, protein, and lactose as a result of infection. Seligsohn *et al*. [43] have studied mastitis in camel dromedarius herds in Kenya. The authors collected milk samples from a minimum of 10 lactating females from each of 20 herds on a daily basis. Bacterial isolates were subcultured on blood agar before being identified using MALDI-TOF. *Streptococcus agalactiae*, *S. aureus*, *Staphylococcus simulans*, *Staphylococcus delphini*, *Staphylococcus rostri*, *S. chromogenes*, *S. hyicus*, *S. haemolyticus*, and *S. warneri* were observed among the samples. de Oliveira *et al*. [44] used MALDI-TOF and 16S sequencing to demonstrate that *Nocardia farcinica* can cause bovine mastitis in Brazil. This result was the initial documented case. Isolates collected from milk samples were also tested for antimicrobial resistance profile, with results suggesting multidrug resistance patterns. Pumipuntu [45] has reported on an emerging species named *Staphylococcus argenteus* which can cause bovine mastitis. The organism is particularly difficult to diagnose, as it is oft misidentified as *S. aureus* by conventional methods. Pumipuntu assessed whether MALDI-TOF could be used to definitively speciate *S. argenteus* from *S. aureus*. The conclusion was that MALDI-TOF could accurately differentiate the novel species, *S. argenteus*, from *S. aureus*. Thus, MALDI measurements are crucial to differentiate these bacteria. Friman *et al*. [46] investigated outbreaks of mastitis on Finnish dairy farms using MALDI-TOF as an investigative tool. Specifically, *Serratia marcescens* infections was confirmed. Interestingly, the authors’ investigative efforts tracked the source of infection to be contaminated teat dip. The dip itself was free from the bacteria, but it became contaminated at the farm before application to cows after milking. The teat disinfectant contained 0.4% or 4000 ppm. N,n-bis(3-aminopropyl) dodecylamine; however, this was ineffective at neutralizing *Serratia*. Wanecka *et al*. [47] were interested in expanding and perfecting diagnostic toolbox for *Staphylococcus* spp. other than *S. aureus*, which are significant causes of intramammary infections in cattle. The authors directly compared 16S RNA sequencing with MALDI-TOF method, and after expansion of the spectral database, the MALDI method identified 97% of samples correctly compared to only 43% for the 16S method.

Matrix-assisted laser desorption ionization-time-of-flight biotyping has also been used for identification/clarification of the causes of clinical cases or outbreaks and for fundamental research of pathogen transmission and antibiotic resistance. Palm *et al*. [48] led a team from the Pennsylvania Department of Agriculture tasked with investigating the death of a 5-day-old boar goat in West Pennsylvania. Bacteria isolated from liver and urachus of the affected animal were identified as *Erysipelothrix rhusiopathiae* by MALDI-TOF MS. The bacteria, believed to be introduced to the farm by insufficient biocontrols, took a significant toll of a 70% mortality rate from the 2019 kidding season. Thomson *et al*. [49] speculated that humans and animals in proximity (companion animals) may exchange bacteria. Antibiotic-resistant bacteria were of primary interest. To investigate, nasal swabs were taken from animals and their owners and MALDI-TOF was used to assess the prevalence of *Staphylococcus* spp. While the authors report finding antimicrobial-resistant strains, no direct link proving owner-pet transmission was reported. Lorente-Leal *et al*. [50] investigated MALDI-TOF to identify non-tuberculosis mycobacteria. The MS method successfully identified over ¾ of isolates with high confidence. However, the method also misidentified several samples. The authors attribute misidentified samples to an incomplete database of reference spectra. Indeed, the expansion of reference spectra databases is an active area of research for mycobacteria [120].

Paiano *et al*. [51] used MALDI-TOF to rapidly identify bacteria present on the uterine walls of cows. Their focus was developing a better understanding of reproductive performance in dairy cows infected with *Trueperella pyogenes*. Santos *et al*. [52] used MALDI-TOF as a rapid screen/confirmation of the identity of *S. aureus* colonies collected from pigs. Recovered colonies confirmed as *S. aureus* were screened for *mec*A and *mec*C genes – genes encoding for methicillin resistance. Future work may be able to identify biomarkers present in the MALDI mass spectrum which can be used to identify antibiotic-resistant organisms without the need for genetic analysis. Pavone *et al*. [53] used MALDI-TOF to confirm the presence of *Comamonas kerstersii* infection in a young goat. This report is the first which documents an infection in animals by this microorganism. A 7-month-old male goat presented with lethargy, weakness, and anorexia before succumbing from the infection. On postmortem examination, an infectious disease of bacterial origin was suspected. In an exceptional demonstration of the method’s utility, MALDI-TOF was used to identify cultured bacteria collected from the animal. This allowed the diagnostic staff to confirm *C. kerstersii* infection for the 1^st^ time in animals. Without MALDI-TOF’s ability to identify the bacteria, the condition would likely have gone undiagnosed.

Mondo *et al*. [54] used MALDI-TOF as a diagnostic tool to demonstrate, for the 1^st^ time, the isolation of *Klebsiella variicola* in a horse with respiratory disease. A 17-year-old Italian saddle horse presented with respiratory distress and fever at a Veterinary Teaching Hospital. On collection of pleural fluid, and isolation of Gram-negative bacteria, the MALDI method was used to identify the bacteria present. The isolate was identified as *K. variicola* by MALDI and isolates of the bacterial colonies were subsequently tested for susceptibility to various antibiotics. Since infection of *K. variicola* is not widely recognized in the previous literature, the exact condition of the horse would likely have been misdiagnosed or not specified. Again, using MALDI-TOF as a diagnostic tool was crucial in developing an improved understanding of the underlying condition.

Finisterra *et al*. [55] have studied whether commercially available dog food in Portugal might be a vehicle for the delivery of antibiotic-resistant *Enterococcus*. To assess this potential hazard, the authors collected 55 samples from commercial brands and susceptibility was studied for 13 antibiotics through the disk diffusion method. Matrix-assisted laser desorption ionization-time-of-flight was used to confirm isolates that were *Enterococcu*s. Feucherolles *et al*. [56] are interested in the surveillance of human and veterinary *Campylobacter* infections. Toward this goal, a strategy must be developed for rapidly identifying isolates for follow-up sequencing. The authors report that MALDI-TOF is a suitable method for the rapid screening of samples for follow-up confirmation by sequencing. Tang *et al*. [57] recovered 284 anal swabs from various animals in two cities in China (Jinhua City and Taizhou). After bacteria were cultured on selective media, the presence of *E. coli* and *Enterococcus* spp. was confirmed with MALDI-TOF. Then, antimicrobial resistance was confirmed in isolated samples, with >80% of isolates exhibiting resistance to tetracycline, sulfamethoxazole, and tamoxifen. Such high rates of antibiotic-resistant organisms highlight concerns over judicious use of antibiotics.

Nguema *et al*. [58] used MALDI-TOF to rapidly type isolates collected from wildlife in Gabon. Isolated colonies were subjected to testing for antimicrobial susceptibility. In addition to MALDI empowering the typing/prevalence of bacterial strains, the authors were able to establish that wild-type isolates carried only intrinsic resistance to antibiotics rather than acquired resistance. Turtles have the reputation of being important carriers of *Salmonella*; however, Doden *et al*. [59] used MALDI-TOF to challenge this notion. In their experiments, the authors swabbed 341 free-range eastern box turtles and enriched them on differential media. Matrix-assisted laser desorption ionization-time-of-flight was used to identify *Salmonella* at the genus basis. Interestingly, only 1.96–3.2% of animals tested positive for *Salmonella*, yielding the authors to conclude that free-range turtles play a minor role in spreading *Salmonella*. Unsatisfied with commercial MALDI spectral databases, researchers from Japan have built their own spectral database relevant to equine bacteria [60]. The authors trained the library using 271 isolates collected from horses and identified the strains through 16S rRNA gene sequencing. The enhanced database increased the number of isolates identified and the average identification score compared with using only the commercial library.

While *Lactococcus garvieae* is most frequently associated with being a fish pathogen, this organism can also cause issues in humans, cattle, sheep, goats, pigs, water buffalos, camels, turtles, snakes, and even domestic pets such as dogs and cats. Thiry *et al*. [61] decided to isolate *L. garvieae* from a nasal swab of a dog and compare the isolates properties with previously sequenced human and animal isolates. Matrix-assisted laser desorption ionization-time-of-flight was used for confirmation of the identity of *L. garvieae*. Interestingly, the isolate was found to be most like one recovered from an Australian camel and an Indian fish.

Brodard *et al*. [62] have used MALDI-TOF to identify strains of *Treponema* spp. which frequently cause digital dermatitis in cattle. Digital dermatitis is a painful infection of the hind hoof, on occasion leading to lameness in the animal and significant economic and animal welfare consequences. The authors report an improved method for culturing the three main strains of *Treponema* spp. (*Treponema pedis*, *Treponema phagedenis*, and *Treponema medium*) from bovine foot specimens on selective growth media before using MALDI-TOF for identification.

Nguyen *et al*. [63] utilized MALDI-TOF to study bacterial causes of abortion of porcine fetuses, specifically those caused by *A. viridans*. Of 103 samples tested, a total of 16 isolates were identified as *A. viridans* by MALDI-TOF. The study provided initial insight into the prevalence of *A. viridans-*induced abortion in pigs located on domestic farms in Korea. Zhu *et al*. [64] noticed a disease-causing severe skin and soft-tissue infection in raccoon dogs in Shandong Province, China. On isolating the bacteria responsible, MALDI-TOF was used to identify the pathogenic organism as *Staphylococcus pseudintermedius*. The author’s note that the isolated strain was capable of infecting mice and warned of great economic loss and the potential zoonotic risk caused by *S. pseudintermedius*.

Celebi *et al*. [65] analyzed an isolate from the blood of a lesser shrew that was captured in the Bartin region of Northwest Turkey. Matrix-assisted laser desorption ionization-time-of-flight indicated *Bartonella*; however, species could not be determined through MALDI-TOF. Follow-up experiments determined that the isolate was a novel species of *Bartonella* that the author’s name *Bartonella refiksaydamii*. Wang *et al*. [66] studied the colistin resistance profile of *Klebsiella* isolated from companion animals. Such a sample has clear zoonotic potential to cross species boundaries and affect humans. In this study, the species of *K. pneumoniae* isolates were determined by MALDI-TOF and confirmed through 16S rDNA sequencing. Specifically, colistin resistance was studied in these samples. Pimenta *et al*. [67] used MALDI-TOF to confirm the identity of isolates as *Staphylococcus* spp. before testing for b-lactam resistance in samples isolated from broiler chickens.

Chanayat *et al*. [68] identified *Staphylococcus* species using MALDI-TOF. They followed up these characterizations with antimicrobial susceptibility testing by the disk diffusion method. The major results of the paper were that of the 65 clinical samples tested, 56 (86.2%) staphylococcal infections were identified with 12/56 (21%) isolates which were MRS infections in dogs with superficial pyoderma. Salgado-Caxito *et al*. [69] confirmed *E. coli* in feces from Brazilian dogs using MALDI-TOF before studying cephalosporin resistance in the bacterial isolates. The rapid confirmation of *E. coli* allowed the authors the ability to delineate risk factors for antibiotic resistance. Not surprisingly, it was found that dogs previously treated with antibiotics were more likely to carry genes associated with antibiotic resistance. In addition, contact with livestock increased odds of antibiotic resistance in bacteria, while dogs previously dewormed were less likely to carry fecal *E. coli* resistant to cephalosporins. Giacon *et al*. [70] used MALDI-TOF for a particularly innovative application. They conducted an experiment to assess risks of infection at a veterinary medical teaching hospital by measuring microbes cultured from air samples. Matrix-assisted laser desorption ionization-time-of-flight measurement identified 29 bacteria at the genus level and 10 bacteria at the species level. Several isolates collected demonstrated multidrug resistance against erythromycin, cephalothin, vancomycin, ampicillin, and ceftazidime. Machado *et al*. [71] focused on the specific issue of subclinical bacteriuria in dogs. The authors sampled urine from dogs and cultured it for bacterial growth. Positive samples were identified by MALDI-TOF. Results found that *E. coli* was responsible for over a third of all cases of infection and MALDI-TOF was a useful tool for the diagnosis of bacterial infection in the canine urinary tract.

Matrix-assisted laser desorption ionization-time-of-flight with database searching represents a powerful investigative tool. For instance, Niedringhaus *et al*. [72] investigated the cause of a multispecies mortality event which occurred in 2018 off the coast of Marco Island, FL. During the outbreak, many birds were found dead, while others were found weak with neurological deficiencies. Necropsy revealed organ inflammation and necrosis associated with a Gram-negative bacterium. Isolation of the bacteria from heart and liver tissues allowed identification through MALDI-TOF measurements. A match for Bisgaard taxon 40 from the *Pasteurellaceae* family was returned, with infection-induced sepsis as the likely cause of mortality. This effort highlights MALDI-TOF as a tool for forensic veterinary science.

Hille *et al*. [73] used MALDI-TOF with advanced software to classify bacteria as not only *Moraxella bovoculi* but also to determine whether the strain produces the so-called repeats-in-toxin (RTX) toxin. This bacterium produces bovine pinkeye, while the RTX toxin is a virulence factor associated with several veterinary pathogens. The authors’ used previous knowledge that the presence of calcium in growth media is required for RTX activity and postulated that calcium may be a limiting factor in the production of RTX for *M. bovoculi*. This premise provided a simple means to vary/control RTX production in cultured cells. Thus, when MALDI-TOF spectra were recorded, it became easier to observe differences in signals between RTX positive and negative groups. Commercially available software (ClinProTools 3.0 software, Bruker Billerica) was used to uncover differences between spectra which can be used as biomarkers. Schauer *et al*. [74] collected nasal swabs from 723 cattle, calves, goats, sheep, llamas, and alpacas were collected in Vienna, Austria. Isolates of staphylococci or mammaliicocci grown on selective media were obtained and species were verified using MALDI-TOF. Confirmed samples were then screened for antibiotic resistance and 158 out of 189 isolates showed phenotypically a multiresistance profile.

Raccoons are an invasive species in Europe and known carriers of *E. coli* in North America. Orden *et al*. [75] wished to determine whether raccoons are carriers on the European continent. To investigate, they collected feces from euthanized animals and were able to obtain 237 *E. coli* isolates and confirmed these by MALDI-TOF. Follow-up experiments determined approximately half of samples contained antimicrobial-resistant *E. coli*. In another manuscript describing microbial sleuths, Bjöersdorff *et al*. [76] collected fecal deposits from trashcans in nine public gardens. *Clostridioides difficile* was isolated through selective plating and MALDI-TOF used for confirmation of presence of the bacteria. Follow-up testing for susceptibility to seven antibiotics was conducted. Fortunately, only approx. 5% of samples contained *C. difficile* and no resistance to metronidazole or vancomycin was detected. Kiiti *et al*. [77] presented a cross-section study to examine antimicrobial resistance profiles in *E. coli* isolates obtained from broiler and layer chickens in Tanzania. Matrix-assisted laser desorption ionization-time-of-flight was used to confirm the presence of *E. coli* in 204 isolates which were subsequently tested for antimicrobial resistance by the disk diffusion method. All isolates tested resistant to ampicillin, and over 85% of isolates were multidrug resistant. Results of this article highlight the very high levels of resistance to commonly used antibiotics used in veterinary and human medicine.

dos Santos *et al*. [78] presented a case study of how MALDI-TOF was used to confirm infection of *S. pseudintermedius* in a bitch. After the infection was noted, enrofloxacin was prescribed; however, within 2 weeks, the dog succumbed to infection. Postmortem analysis determined that isolates of *S. pseudintermedius* recovered from the dog were resistant to 16/19 antibiotic protocols tested. da Silva *et al*. [79] trained biotyping software on MALDI-TOF spectral data to identify *Brucella canis* and differentiate it from a closely related *Brucella suis*. While the paper was mainly focused on canines in Brazil, the method could presumably be used on isolates collected from a variety of species for this zoonotic organism. The work highlights both the ability of MALDI-TOF to rapidly identify causes of outbreaks but also achieve definitive diagnosis of challenging conditions in a high-throughput format. Uchida-Fujii *et al*. [80] from the equine research institute in Shimotsuke, Japan, had an interest in using MALDI-TOF biotyping to rapidly identify bacteria isolated from horses. The authors isolated 3724 bacterial isolates taken from horses over the period of 1980–2016 and recorded mass spectra on these samples after growth on Columbia agar plates. These authors note that incomplete spectral databases limit the utility of MALDI-TOF for biotyping. Despite 86.2% of isolates being identified to the species level, the authors note some limitations of the spectral libraries as major equine pathogens such as *Taylorella equigenitalis* and *Rhodococcus equi* proved difficult to identify.

Matajira *et al*. [81] performed bacterial identification by MALDI-TOF MS. *Vagococcus lutrae* and *Vagococcus fluvialis* were confirmed present in swine located in Brazil. Pipan *et al*. [82] performed an interesting experiment in which the microbiome of newborn puppies was investigated and compared with the maternal vaginal and oral microbiome. Bacterial colonies were identified by MALDI-TOF. Results indicated that puppy meconium microbiota resembled bacteria from the maternal vagina when puppies were delivered vaginally. However, when cesarean birth occurred, the meconium was more like a blend of the oral and vaginal biota. Interestingly, most placental samples contained bacteria of multiple genera – challenging the sterile womb theory. In addition, it was found that puppies born without an established meconium microbiota demonstrated a slower growth rate after birth. In another work, *Staphylococcus* isolates collected from various species over a 10-year period from a Veterinary Teaching Hospital of Complutense University of Madrid were identified using MALDI-TOF biotyping before the study of antibiotic resistance [83]. The authors found a high prevalence of multidrug resistance and resistance to fluoroquinolones, cephalosporins, and macrolides. Infectious synovitis is often caused by *Mycoplasma* infection in poultry flocks. Such infection can cause upper respiratory infections, reduced growth, production, and lower egg hatchability rates.

In the work of Cisneros-Tamayo *et al*. [84], MALDI-TOF was used to screen poultry flocks for *Mycoplasma* and it was found that *Mycoplasma synoviae* was detected in 25/28 flocks tested while *Mycoplasma pullorum* was observed in 56% of flocks considered. In addition to providing data on prevalence, the paper demonstrates the use of MALDI-TOF technology for rapid screening of poultry flocks for *Mycoplasma* infection. In another work considering antimicrobial resistance, Sukmawinata *et al*. [85] collected fecal samples from 212 healthy racehorses in Japan and cultured collected material on *Enterococcus* selective medium. *Enterococcus* isolates were confirmed using MALDI-TOF. Then, antimicrobial susceptibility tests were conducted against 11 antimicrobials, including ampicillin, vancomycin, streptomycin, gentamycin, kanamycin, oxytetracycline, chloramphenicol, erythromycin, lincomycin, tylosin, and enrofloxacin. The authors found antimicrobial resistance rates between 0.5% and 50% for the various substances tested, with enrofloxacin being the most common.

Moffat *et al*. [86] reported on antibiotic resistance noted in Turkey. They obtained pooled fecal samples collected from 77 Turkey farms in British Columbia, Quebec, and Ontario and identified *Enterobacteria* after culture. The authors note that approx. 93% of positive samples were identified as *E. coli* and only a few other species of *Enterobacterales* were identified. The authors also screened for several antimicrobial resistance genes (*bla*CMY, *bla*CTX-M, *bla*TEM, and *bla*SHV). Fortunately, the prevalence of antimicrobial resistance in the samples was roughly only 5%. Of the antimicrobial-resistant samples, about 71% possessed the gene responsible for resistance to ceftriaxone. Rapid identification of *T. pyogenes* is important as this organism causes mastitis, abortion, and infections of the reproductive tract in livestock, including cattle, sheep, goats, horses, and pigs.

Ahmed *et al*. [87] used MALDI-TOF for identifying *T. pyogenes* isolates recovered from a vaginal discharge of an okapi (*Okapia johnstoni*) and the kidney of a female royal python. Shah *et al*. [88] reported on an emerging One Health concern – the growing popularity of backyard poultry flocks in the United States and corresponding increased risk of human-animal contact and transmission of pathogens. In this work, 34 residential flocks of poultry were sampled for *Salmonella* prevalence and multidrug-resistant Gram-negative bacteria. Similar to other works, the authors used MALDI-TOF to identify bacterial isolates. The authors then studied antimicrobial resistance profiles using the disk diffusion method. Results revealed that *E. coli* was detected in approx. 2/3 of flocks, while *Salmonella* was present in 3%. *Acinetobacter* and *Pseudomonas* strains were also noted presently.

Frye *et al*. [89] used MALDI-TOF to diagnose *Clostridium botulinum* type A in dairy cattle in New York state during an outbreak. Isolates were collected from the rumen and liver of deceased animals and confirmed using MALDI-TOF. The bacterial spores can be stable for years in the environment and can release potent neurotoxins, which are extremely damaging to cattle. In this outbreak, nearly half of the affected cattle died, with the remaining fraction never returning to full productivity. The authors identified peptide biomarkers at m/z = 2406 and daughter ions resulting from cleavage of the BoNT/A peptide present at m/z = 998 and 1426. The presence of these ions can be used as biomarkers for rapid diagnosis of botulism in cattle.

In an interesting study of clinical veterinary relevance, Lavallee *et al*. [90] used MALDI-TOF to study the microbiome of canine arthrocentesis sites. The authors focused on how aseptic skin preparation techniques affected bacterial counts, and whether clipping the animals’ hair before the procedure affected results. *Staphylococcus* spp. were the most common bacterial species cultured after aseptic cleansing. It was found that hair clipping for aseptic site preparation was not crucial for aseptic site preparation. The research project provides empirical evidence to drive best practices in the veterinary surgical procedure.

Once a core diagnostic facility acquires the MALDI-TOF apparatus, the expense per sample is relatively low, with rapid turnaround times. This aspect of MALDI-TOF was highlighted by Medkour *et al*. [91], who used MALDI-TOF to screen bacterial infections in deceased street rats in Marseille, France. *Staphylococcus cohnii*, *Bordetella bronchiseptica*, *Bordetella parapertussis*, *Corynebacterium glucuronolyticum*, *Pelistega suis*, and *Rodentibacter rarus* were confirmed in a rat and this result illustrates the utility of MALDI-TOF being present within a central veterinary diagnostic facility.

Van Damme *et al*. [92] have considered 237 cases/diagnosis of superficial canine pyoderma presenting to a veterinary teaching hospital in the Netherlands. Pyoderma is an infection causing pus-filled lesions in the skin. Isolates of *S. pseudintermedius* collected from the lesions were identified by MALDI-TOF MS. Following isolation/confirmation steps; the authors tested isolates for antimicrobial resistance to methicillin and clindamycin. Results indicated that the prevalence of antimicrobial resistance nearly doubled if a dog had previously been exposed to an antibiotic treatment regimen (37.7% vs. 21.1%). Scherrer *et al*. [93] desired to characterize the genetic diversity of wild-type *Streptococcus suis* collected from swine. This organism is a crucial swine pathogen, with significant zoonotic potential. *Streptococcus suis* can cause skin infections in individuals who handle uncooked pork and has been known cause gastrointestinal infection and even meningitis. Bacterial isolates collected were identified/confirmed as *S. suis* by MALDI-TOF. Genetic analysis and extent of expression of virulence markers were then explored by a variety of techniques.

In addition to reviewing sampling techniques for the collection of samples for clinical diagnosis of bovine respiratory disease, Pardon and Buczinski [94] comment on MALDI as an exciting diagnostic tool for rapid identification of bacterial infections in the future. The authors conclude that as MALDI-TOF instruments become more widespread, the method should be adopted more since the per sample cost is very low. Werinder *et al*. [95] isolated *S. suis* from grower pigs in Sweden and used MALDI-TOF to confirm the identity of the bacteria after culture. Confirmed isolates of the pathogen were then subjected to testing for antimicrobial resistance by the broth microdilution method using commercially available products. A high prevalence of *S. suis* was found as isolates could be recovered from ~95% of pigs tested. Although only 3.8% of isolates were resistant to penicillin, tetracycline resistance was common (88.4%).

Carvalho *et al*. [96] have provided insights into the role of companion animals spreading beta-lactamase and carbapenemase-producing *K. pneumoniae* isolates. In this work, MALDI-TOF was used to identify *K. pneumoniae* collected from fecal samples from 356 dogs. In all, only 4.4% of samples were positive for this pathogen; however, approx. 94% of the positive samples carried genes for expression of beta-lactamase. The results suggest that dogs can be important carriers of extended-spectrum beta-lactamase-producing *Klebsiella*. *Mycobacterium bovis* is a major cause of pneumonia in calf operations, causing millions of dollars of losses annually. Bokma *et al*. [97] desired to use MALDI-TOF methods as a rapid screening tool for diagnosis of *M. bovis* infection from bronchoalveolar lavage fluid. The typical *M. bovis* concentration in lavage fluid usually ranges from 10^3^ to 10^8^ CFU/ml, so adequate numbers of cells are present for analysis. After 24 h of growth in enrichment broth, the MALDI method suggested a prevalence of <3%. However, the observational prevalence increased to 30–38% when 48–72 h of enrichment was carried out. Real-time polymerase chain reaction (PCR) and biochemical testing for lipase activity both suggested that 28/104 samples were positive for *M. bovis* with several other samples inconclusive. Thus, reasonably good agreement between the MALDI methods and reference methods was achieved.

Bovine pinkeye (keratoconjunctivitis) is often caused by *Moraxella bovoculi*. Interestingly, two genotypes of this organism have been discovered – genotype 1 and genotype 2. Both genotypes have been isolated from the eyes of cattle not presenting with clinical signs of pinkeye. However, only genotype 1 has been observed in clinical cases. Thus, Hille *et al*. [98] developed a MALDI-TOF approach to genotype *M. bovoculi* strains using mass spectrum biomarkers. Despite similarities between genotypes, the authors report that accuracies ranging from 90.6% to 100% can be achieved. Thus, MALDI-TOF can be a useful tool in screening cattle for the presence of the more hazardous clinically relevant version. Holmström *et al*. [99] tackled the issue of prevalence of methicillin-resistant *S. pseudintermedius* in companion animals (dogs and cats). Isolates obtained from clinical samples were confirmed as isolates of *S. pseudintermedius* using MALDI-TOF. Methicillin-resistance detection was carried out on isolates, as was direct detection of the *mecA* gene. Results provide important insights for veterinary professionals worldwide. It was found that a majority of *S. pseudintermedius* from canine samples were obtained from infectious processes on the skin, ear, and urinary tract. However, in cats, there was a prevalence of this pathogen in urinary tract infections.

Brockmann *et al*. [100] worked to evaluate the range of aerobic bacteria in skin lesions of reptiles and to determine their antimicrobial susceptibility by culturing swabs collected from 219 reptiles. Isolates were grown on selective agar plates before being identified using MALDI-TOF. Over 300 isolates were identified, including specimens of *Pseudomonas* spp., *Citrobacter* spp., aerobic spore-forming bacteria, *Aeromonas* spp., *Acinetobacter* spp., *Proteus* spp., *Staphylococcus* spp., *Klebsiella* spp., *Enterococcus* spp., and *Morganella* spp. as well as 78 other Gram-negative and 12 other Gram-positive bacteria. The versatility of MALDI for biotyping is on display in this work, allowing the authors to create a valuable survey of the microbiome of reptile skin.

Alssahen *et al*. [101] used MALDI-TOF to identify nine *Arcanobacterium phocae* strains isolated from cases of mink dermatitis in Finland. The study highlights the remarkable versatility of MALDI for veterinary diagnosis. Eltawab *et al*. [102] used MALDI-TOF biotyping to study *Brucella* spp. circulating in bovine and buffalo dairy herds in the Nile delta region of Egypt. One hundred samples were collected from placentas, vaginal swabs, uteri, mammary lymph nodes, and stomachs of aborted fetuses. The overall prevalence in cattle ranged from 10.8% to 66.6% and was highly variable with tissue type. A limited number of buffalo samples exhibited a prevalence of roughly 50%. The survey data provide insights into brucellosis in Egypt. In 2010, 10 ewes and eight lambs of a flock in Kosovo exhibited fever, facial paralysis, and a host of additional neurological maladies. Several members of this cohort died within a week of onset. After death, the brainstem of the animals was excised for analysis. *Listeria* spp. was confirmed in these samples using MALDI-TOF [103]. The authors followed up the confirmation by assessing antibiotic resistance profile. *Listeria monocytogenes* isolates collected were susceptible to penicillin, erythromycin, tetracycline, streptomycin, trimethoprim/sulfamethoxazole, quinupristin/dalfopristin, kanamycin, vancomycin, and gentamicin and resistant to nitrofurantoin and lincomycin.

Brown *et al*. [104] used MALDI-TOF to solve a mysterious outbreak on a New Zealand farm in which 15 calves became ill. Dead calves were found to have lung lesions, and microscopy revealed rod-shaped bacteria in samples. Isolates were subjected to MALDI-TOF analysis and were subsequently identified as *Bibersteinia trehalosi* in the lung. Moroz *et al*. [105] desired to establish the prevalence of nasal cavity staph infection among the Polish goat population. Toward this goal, 1300 nasal swabs were obtained from clinically healthy goats in the summer of 2014. The swabs were incubated in Mueller-Hinton broth for 24 h. Isolates were subjected to MALDI-TOF using CHCA matrix before identification as *Staphylococcus*. Matrix-assisted laser desorption ionization-time-of-flight confirmed the presence of *Staphylococcus* spp. in 437/1300 samples (29.1%). Achek *et al*. [106] reported on the diversity of organisms causing mastitis in sheep in Algeria. In brief, 123 milk samples of sheep presenting with mastitis were collected and staphylococci were isolated using selective growth media. Recovered isolates were then subjected to MALDI-TOF analysis for confirmation of identity. Out of 123 sheep milk samples, 41 were positive for staphylococci (33.3%). Of the positives, almost 2/3 were identified as MSSA.

Ulrich *et al*. [107] evaluated whether a popular test kit (Sepsityper, Bruker Billirica) to detect human sepsis through MALDI-TOF also works for canine and feline blood samples. In the experiments, the authors inoculated authentic blood samples at two concentrations (10 and 100 cfu/mL) with bacteria which are common causes of animal sepsis. The authors report that all inoculated samples tested positive, while control samples were negative for bacterial growth. In addition, MALDI-TOF identified all 72 samples tested as exact matches to results obtained by conventional microbiological analysis. The authors conclude that the MALDI Sepsityper kit marketed by Bruker can work with feline and canine blood samples. This technology offers the advantage of being approx. 24 h faster than conventional methods for sepsis diagnosis. This rapid turnaround enables clinicians to treat cases more rapidly, saving valuable time before advancement of the condition. Van Driessche *et al*. [108] addressed a significant problem with using MALDI-TOF as a diagnostic tool – the time required for culturing bacteria prior to identification. In this work, the authors optimized bacterial growth conditions to achieve sufficient cellular material for MALDI in only 6 h. Consequently, a turnaround time of <1 working day was achieved for bronchoalveolar lavage fluid samples. The development could be significant for the diagnosis of bovine respiratory disease. Infections caused by *Bergeyella zoohelcum* after animal bites can present serious human clinical disease.

Muramatsu *et al*. [109] aimed to better constrain the risk of *Bergeyella* infection by surveying the prevalence of this bacteria in the oral cavity of therapy dogs. Matrix-assisted laser desorption ionization-time-of-flight was used for bacterial identification. Of 150 animals screened, 20 animals produced samples which were positive for *B. zoohelcum*. The authors report that the MALDI method developed is the first of its kind for screening for *Bergeyella* in canines. Nisa *et al*. [110] were interested in identification *S. pseudintermedius*, known to colonize the integument of dogs. The authors obtained 117 samples from dogs and cats in New Zealand (106 dogs and 11 cats) collected between 2014 and 2017. Bacterial samples of approx. 2 mm^3^ volume were subjected to full protein extraction by homogenization in 300 μL of water followed by 900 μL ethanol before spotting on MALDI plates. The authors conclude that sensitive and specific species-level identification of *S. pseudintermedius* can be achieved using MALDI-TOF, and the method is a valuable veterinary diagnostic tool.

Rau *et al*. [111] were interested in studying the zoonotic organism *Corynebacterium ulcerans*, which causes skin abscesses in several animal species. However, toxigenic strains of *Corynebacterium diphtheriae* group can also cause diphtheria. Thus, they aimed to establish a MALDI-TOF MS method for reliable speciation between various members of *C. diphtheriae* group. The authors collected reference mass spectra for known samples to add to their MALDI spectral library. As a result, the success of identification at species level was increased from 88.3% to 100% for the 103 *C. ulcerans* isolates tested.

In their work, Bokma *et al*. [112] realized the potential of MALDI-TOF for diagnosis of *M. bovis* infection. However, they identify a key bottleneck in the workflow – the need to grow/incubate bacteria before successful analysis. In this work, growth conditions and incubation time were investigated to optimize identification of *M. bovis*. The authors report that under optimal conditions developed, reliable identification of *M. bovis* with MALDI-TOF was possible for 83% of samples as early as 24 h after inoculation.

Wickhorst *et al*. [113] used MALDI-TOF to identify *Trueperella abortisuis* in companion animals (dogs and cats) in Germany. Previously, this organism was observed only in livestock. The work demonstrates conclusively that this organism can be found in pets and thus poses a potential for transmission to humans. Baudler *et al*. [114] reported that diagnosis of avian *Mycoplasma* by conventional methods can be a time-consuming task fraught with difficulty. Hence, these authors developed a MALDI-TOF *Mycoplasma* spectral database of 36 main spectrum profiles from 23 avian *Mycoplasma* spp. reference strains, one live vaccine strain, and eight clinical isolates. Then, to test the ability to identify clinical strains, 112 clinical isolates were tested and it was found that 96% of samples were identified definitively at the species level. Of the remaining samples, 80% provided tentative matches for *Mycoplasma* spp.

Becker *et al*. [115] set out to validate a free, online spectral database of over 900 species of fungi for isolates sourced from veterinary laboratories. Of 290 isolates analyzed, 258 (89% of the total isolates), representing 47 different species, were correctly identified at the species level using MALDI-TOF. This performance was far greater than determination based on morphologic traits and physiological tests, which were correct at the species level only 60% of the time.

Despite the successes of MALDI-TOF for veterinary diagnosis, the work of Schlez *et al*. [121] highlights limitations of MALDI-TOF and the need for genomic analysis for definitive identification. In this work, MALDI-TOF analysis identified bacterial isolates collected from three separate animal cases as *C. diphtheriae*. It turns out that the key spectral features in the MALDI mass spectrum are highly similar to spectra of *C. rouxii*. The latter organism was confirmed after whole genome sequencing. Bokma *et al*. [122] have reported that mass spectral peaks caused by growth media (agar) can lead to false-positive results for the identification of *Mycoplasma alkalescens* and *Mycoplasma arginini*. The authors identified several peaks between m/z = 3000 and 4500 peaks originating from pleuropneumonia-like organism Aagar, horse serum, and colistin which are presumably responsible for the false positives. These works highlight the need to confirm conclusions made with MALDI-based methods and highlight the need for operators to understand the MALDI technology and its limitations.

## Matrix-assisted Laser Desorption Ionization-time-of-flight for Entomology/Parasitology/Fungal Infections

While MALDI-TOF measurements are most frequently associated with the identification of isolated bacteria after culture, investigators continue to push the limits of the technique and explore applications in additional diagnostic realms. Table-2 summarizes many of these emerging applications of MALDI-TOF [123–136]. Fungal diseases of the dermis represent a common class of clinical presentations. At present, identification of organisms with an affinity for keratinized tissue (dermatophytes) occurs by observation of morphology or molecular methods. These diagnoses are costly and require significant time, so most clinicians may not even bother to recommend a diagnosis given the obstacles. Recognizing the opportunity to close the gap, Chen *et al*. [123] conducted a meta-analysis of the existing literature. These authors identified 15 recent studies which considered nearly 2000 dermatophytes. The analysis found that MALDI-TOF is an accurate approach to identify dermatophytes, with an identification ratio of 0.96 (95% confidence interval [CI] = 0.92–1.01) and 0.91 (95% CI = 0.86–0.96) at the genus and species level, respectively. However, the technique is still limited by culture time required before MALDI measurement (at least 7 days as reported). Yeasts also represent an additional class of dermal conditions requiring rapid confirmation. Lara *et al*. [124] evaluated MALDI-TOF for the identification of *Trichosporon* spp. and compared results with phenotypic and genotypic typing. The paper reports an agreement of 55.9% between phenotypic and molecular methods, while MALDI classified 74.5% of samples as genus *Trichosporon* yeasts. In another interesting article, Hernandez-Bures *et al*. [125] used MALDI-TOF to screen stray dogs and cats in Puerto Rico for dermatophytes. Dermatophytosis in stray animals may reach a prevalence of 50%; however, the current paper found that only roughly 20% of animals were infected. A total of 19 animals (19%) were positive for dermatophyte growth. Of these animals, 18/19 were infected with *Mycoplasma canis* and 1/19 with *Trichophyton* spp. Matrix-assisted laser desorption ionization-time-of-flight was critical for making this diagnosis. Awandkar *et al*. [116] used MALDI-TOF to confirm *Candida* isolates associated with cases of bovine mastitis. The work confirmed *Candida albicans* as a significant fungal agent involved in clinical bovine mastitis and identified *Kodamaea ohmeri* isolates recovered from bovine clinical mastitis as an opportunistic emerging pathogen. Baumbach *et al*. [126] utilized MALDI-TOF to identify dermatophytes that can cause inflammatory skin lesions. Rapid and accurate diagnosis is required for a veterinarian to initiate targeted and effective therapy. While the unmodified MALDI-TOF spectral database did not return matches or hits for 50 clinical samples of hedgehogs tested, the authors did not relent. Instead, they sequenced DNA of isolated specimens and added MALDI-TOF spectra to the searchable database. This allowed acceptable identification of *Trichophyton erinacei* and *Arthroderma* spp. in samples collected. The dermatophyte *Microsporum canis* can infect both animals and humans. Conventionally, diagnosis relies on the skill of the technician to identify the species. Although, more recently, molecular methods (DNA analysis) have been emerging as a viable alternative. Toward improving diagnosis, Hariu *et al*. [127] recorded MALDI mass spectra for *M. canis* isolated from an infected family in Japan. The authors determined that MALDI-TOF biotyping was an effective and cost-effective alternative for diagnosis of this dermatophyte.

**Table-2:** List of recent application of MALDI-TOF for diagnosis of insects or fungal infection.

Species identified	Notes	References
Various described	Meta-analysis, concludes MALDI-TOF, is suitable for dermatophyte identification	[123]
*Trichosporon* spp.	MALDI classified the correct genus approx. 75% of samples	[124]
*M. canis, Trichophyton* spp., various	Prevalence and speciation from stray animals in Puerto Rico	[125]
T. *erinacei* and *Arthroderma* spp.	Inflamed skin lesions – hedgehogs	[126]
*M. canis*	Sample from infected family in Japan	[127]
*T. spiralis, T. britovi, T. nativa, T. pseudospiralis*, and *T. patagoniensis*	MALDI-TOF can be used to speciate *Trichinella*. The authors used known reference strains to create 128 main reference spectra for the identification	[128]
*Nannizziopsis guarroi*	Developed MALDI diagnostic for parasite affecting reptiles	[129]
*Nosema bombycis*	Developed MALDI method for rapid screening for organism	[130]
*Cimex hirundinis*	Heads or thorax of bugs were dissected, extracted and ran on MALDI-TOF	[131]
*Cimex lectularius* and *C. hemipterus*	Trained spectral library, achieved high % matching	[132]
10 *Culicoides* species	12 animal farms throughout Israel, dissected the wings off, and used the abdomen and thorax for MALDI-TOF analysis	[133]
Various *Culicoides* species	Sampled at variety of locations in Switzerland	[134]
Lice including *B. ovis, B. bovis, B. caprae, L. vituli, L. africanus, H. eurysternus, S. capillatus, C. meleagridis, G. gigas, M. stramineus, M. gallinae, G. gallinae, L. caponis*, and *P. humanus corporis*	Identifying lice collected from livestock and poultry in Algeria	[135]
Various tick species	Built spectral library, concluded that MALDI-TOF is a viable alternative for tick identification	[136]

MALDI-TOF=Matrix-assisted laser desorption ionization-time of flight, *M. canis*=*Microsporum canis,*

*T. erinacei*=*Trichophyton erinacei, T. spiralis*=*Trichinella spiralis, T. britovi*=*Trichinella britovi, T. nativa*=*Trichinella nativa, T. pseudospiralis*=*Trichinella pseudospiralis, T. patagoniensis*=*Trichinella patagoniensis, C. hemipterus*=*Cimex hemipterus, B. ovis*=*Bovicola ovis, B. bovis*=*Bovicola bovis, B. caprae*=*Bovicola caprae, L. vituli*=*Linognathus vituli, L. africanus*=*Linognathus africanus, H. eurysternus*=*Haematopinus eurysternus, S. capillatus*=*Solenopotes capillatus, C. meleagridis*=*Chelopistes meleagridis, G. gigas*=*Goniodes gigas, M. stramineus*=*Menacanthus stramineus, M. gallinae*=*Menopon gallinae, G. gallinae*=*Goniocotes gallinae, L. caponis*=*Lipeurus caponis, P. humanus corporis*=*Pediculus humanus corporis*

**Figure-3:**
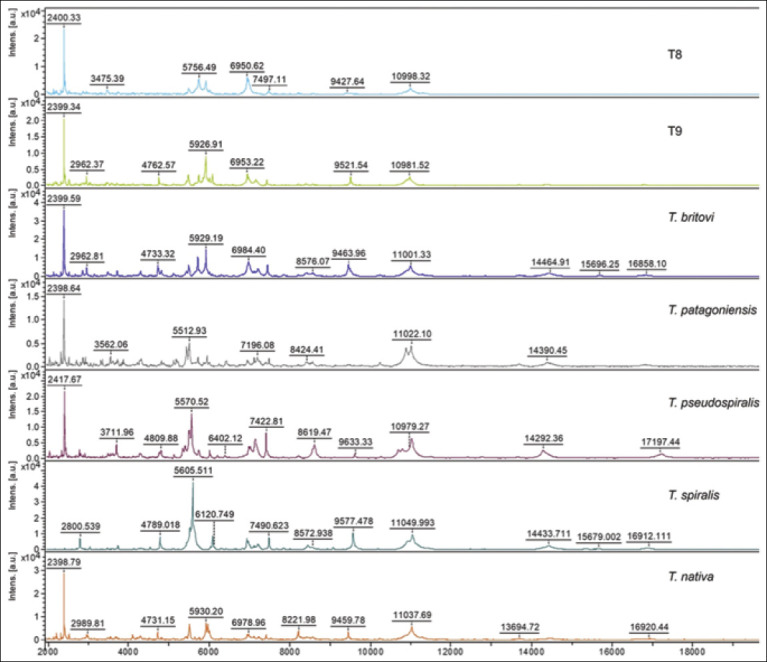
Matrix-assisted laser desorption ionization time-of-flight spectra of *Trichinella* samples. The m/z for *Trichinella* T8 (a), *Trichinella* T9 (b), *Trichinella britovi* (c), *Trichinella patagoniensis* (d), *Trichinella pseudospiralis* (e), *Trichinella spiralis* (f), and *Trichinella nativa* (g) are given. Differences in peaks are noted between samples allowing identification. Figure reproduced from Karadjian *et al*. [128] under license from Elsevier.

When *Trichinella* larvae are discovered during a routine examination, there arises a need to identify/diagnose at a species level. Molecular biology methods (PCR tests) are typically used for gold standard diagnoses; however, Karadjian *et al*. [128] challenged this notion and suggested that MALDI-TOF can be used to speciate this parasitic infection. The authors used known reference strains to create 128 main reference spectra for the identification library which included samples of *Trichinella spiralis*, *Trichinella britovi*, *Trichinella nativa*, *Trichinella pseudospiralis*, and *Trichinella patagoniensis*. The authors found that all *Trichinella* species could easily be differentiated by patterns of peaks in the mass spectra (Figure-3) [128]. The authors concluded that MALDI-TOF is a reliable, rapid, easy-to-use, and cheap tool for *Trichinella* species identification.

*Nannizziopsis guarroi* is a fungal pathogen affecting reptiles. Schneider *et al*. [129] commented on the increasing frequency of clinical cases since 2009 and the need for a diagnostic tool for veterinary practices. Toward this goal, the authors developed a MALDI-TOF-based method. In conclusion, the authors acknowledge MALDI-TOF accessible protein profiling as a useful diagnostic tool of reptile-associated pathogenic fungi.

A condition known as Pébrine is a lethal insect disease affecting silkworms and honeybees caused by infection of *Nosema bombycis*. Kajiwara *et al*. [130] wished to develop a reliable means to confirm the presence of this organism and explored MALDI-TOF to accomplish this. The authors infected silkworm larvae by feeding *N. bombycis* at 4.3 × 10^4^ spores/g in artificial diets. Spore proteins were extracted in 70% formic acid using a plastic rod in a tube, before spotting on a MALDI plate and analysis. The authors discovered most prominent peak obtained from *N. bombycis* infected larvae was at m/z 3203.7 and unique MS peaks. The authors concluded that the method could determine an infection and identify *N. bombycis* within 30 min of sample preparation.

**Figure-4:**
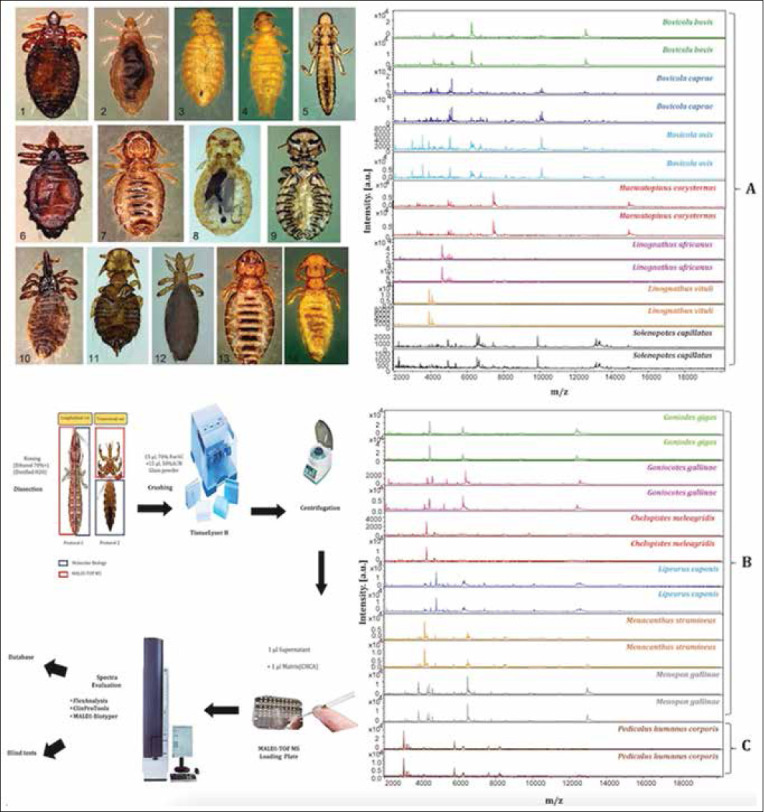
Photographs of 13 species of lice collected from three regions of northeastern Algeria: *Solenopotes capillatus* (1); *Pediculus humanus corporis* (2); *Menopon gallinae* (3); *Menacanthus stramineus* (4); *Lipeurus caponis* (5); *Haematopinus eurysternus* (6); *Bovicola caprae* (7); *Goniocotes gallinae* (8); *Goniodes gigas* (9); *Linognathus vituli* (10); *Chelopistes meleagridis* (11); *Linognathus africanus* (12); *Bovicola bovis* (13); and *Bovicola ovis* (14). Species can often be wrongly identified given similarity. *Bottom Left* – matrix-assisted laser desorption ionization (MALDI) workflow for the identification of lice. Aggressive conditions of 70% formic acid were used for digestion of sample. *Right* – MALDI spectra for a variety of poultry and mammalian lice. Figure reproduced from Ouarti *et al*. [135] under the Creative Commons Attribution License (https://creativecommons.org/licenses/by/4.0), which permits unrestricted use, distribution, and reproduction in any medium, provided that the original work is properly cited.

Another particularly exciting field of research is use of MALDI-TOF for entomology. In principle, insect samples may also exhibit characteristic protein signatures which can be used for species identification. However, sample preparation may be difficult because digestion/extraction of characteristic proteins from insect tissues can prove challenging. Nonetheless, researchers have persisted in this endeavor and have developed sample prep protocols which are effective. Indeed, sample preparation and sample storage conditions are often discussed extensively within the following publications. Hamlili *et al*. [131] extended the field of MALDI-TOF analysis of parasites of veterinary interest by demonstrating overwhelming success in identifying Swallow bugs in France. Swallow bugs were rinsed in 70% ethanol and water before being dried on paper. Either the heads or heads and thorax of the bugs were dissected before being rinsed and transferred to a vial with formic acid and acetonitrile. The tissues were homogenized, and resultant solution was mixed with matrix on a standard MALDI plate. Known specimens were used to build a reference spectral library. Then, blinded samples were run with quality MS spectra being compared to the database. Results indicated that *Cimex hirundinis* nymphs were accurately identified, 100% of the samples at the species level. Benkacimi *et al*. [132] used the benefits of MALDI-TOF analysis for rapid molecular biotyping to identify bedbugs (*Cimex* *lectularius* and *Cimex hemipterus*). This study followed a typical workflow for insects, including washing the bug, dissecting, and homogenizing within a solvent before spotting on a MALDI plate. Between 2 and 5 high-quality mass spectra of laboratory and wild strains of the *Cimex* species were entered into the MALDI database. Then, blind testing of 167 specimens yielded a 100% match at species level and 86.25% match to origin. Rot *et al*. [133] were tasked with developing a diagnostic for rapidly identifying biting midges which serve as biological vectors for numerous pathogens of veterinary significance. Three identification methods – classical morphology, DNA barcoding, and MALDI-TOF, were applied and compared to individuals of 10 *Culicoides* species. The authors trapped *Culicoides* at 12 animal farms throughout Israel, dissected the wings off, and used the abdomen and thorax for MALDI-TOF analysis. For MALDI-TOF protein profiling, the thorax and legs of each insect were homogenized in 20 μL of 10% formic acid, and a 5 μL aliquot mixed with 7.5 μL sinapic acid in 60% acetonitrile, 40% H_2_O, and 0.3% TFA. The MALDI-TOF measurements were able to discern 10 species of Israeli *Culicoides* based on proteomic profiles after database training. Paslaru *et al*. [134] used MALDI-TOF for a unique application – the characterization of the population of biting midges collected in Switzerland. *Culicoides* (Diptera: *Ceratopogonidae*) are small biting flies which are known biological vectors of arboviruses, including bluetongue virus, Schmallenberg virus, and African horse sickness virus. The flies can cause severe allergic dermatitis in horses. Over 1000 individual flies were collected at two locations and identified to species level using MALDI-TOF. The most predominant species sampled at one location were *Culicoides* *obsoletus* (30%) and *Culicoides pulicaris* (20%), while at a second location, the main catches were *Celiptera* *grisescens* II (41%) and *C. pulicaris* (30%). The application of MALDI-TOF to speciation of insects is a remarkable display of the technique’s robust utility. Ouarti *et al*. [135] reported on testing MALDI-TOF for identifying lice collected from livestock and poultry in Algeria. The authors note that morphological identification of lice is very challenging because species are often morphologically very similar. Figure-4 illustrates the challenge posed [133]. To address this issue, the authors worked to optimize sample preparation protocols which resulted in 57 high-quality reference spectra being added to the identification database for species inclusive of *Bovicola ovis*, *Bovicola bovis*, *Bovicola caprae*, *Linognathus vituli*, *Linognathus africanus*, *Haematopinus eurysternus*, *Solenopotes capillatus*, *Chelopistes meleagridis*, *Goniodes gigas*, *Menacanthus stramineus*, *Menopon gallinae*, *Goniocotes gallinae*, *Lipeurus caponis*, and *Pediculus humanus corporis*. The work enables identification of 14 species of lice parasites which previously may have been misidentified due to morphological similarities. In another entomological work, Benyahia *et al*. [137] used MALDI-TOF measurements to correctly identify a variety of species of lice with over 94% effectiveness – even after collected specimens were stored in alcohol. *Hyalomma* species is a common vector for hemorrhagic fever. Rapid identification of these ticks can be performed for intact specimens; however, species discrimination can be difficult for damaged during collection or blood-fed ticks. In recent years, MALDI-TOF has been used for tick identification for samples of leg proteins. Schulz *et al*. [136] used this strategy to build a MALDI spectral library for several tick species. The authors obtained authentic samples before removing four legs of the ticks with a scalpel and transferring them into an Eppendorf tube. For each tick sample, legs were mixed with 20 μL of 70% formic acid and a spatula of glass powder before sonication, shaking, and combination with matrix on the MALDI plate. All 15 tick variants were studied, with reference, MALDI spectra was collected for each. The authors concluded that MALDI-TOF is a viable alternative for tick identification.

## Matrix-assisted Laser Desorption Ionization-time-of-flight for Molecular and Protein Profiling

Despite the popularity of MALDI-TOF for the identification of microorganisms or ticks, it is important to remember the technique itself provides relevant chemical information. Identification of biomarkers or tracking changes in sample chemical components may often signal the presence of physiological conditions requiring treatment regimens. Table-3 summarizes recent applications of MALDI-TOF for diagnostics based on molecular typing [138–154]. For the first example, Riengvirodkij *et al*. [138] used MALDI-TOF in effort to identify peptide biomarkers associated with mitral valve disease in dogs. The results of this study are truly remarkable, with a 3D principal components analysis definitively demonstrating the ability to not only detect mitral valve disease but also specify stage. Six amino acid sequences of peptide candidates at m/z 1225.60, 1363.85, 1688.71, 1789.52, 2020.21, and 2156.42 Da were identified as putative biomarkers for the condition.

**Table-3:** Applications involving chemical profiling for veterinary science using MALDI-TOF.

Molecule of interest	Notes	References
m/z 1,225.60, 1,363.85, 1,688.71, 1789.52, 2020.21, and 2156.42 Da	Biomarkers associated with mitral valve disease in dogs	[138]
Various – see reference	Salivary biomarkers of protein expression present in canine melanoma cases	[139]
Tumor necrosis factor receptor superfamily member 18 (TNFRSF18)	Study differential protein expression between pigs with descended versus undescended testes	[140]
50 unique proteins from *E. necatrix*	Identify proteins characteristic of this organism which causes reduced production in poultry operations	[141]
Immunoprotective proteins between 15 kDa and 100 kDa	Discover three new immunogenic proteins which were protective against porcine *Pasteurella multocida*	[142]
Identified proteins expressed during immune reaction to *Brucella*	Developed a sensitive and specific enzyme-linked immunosorbent assay test for *Brucella* infection in humans	[143]
Succinyl-diaminopimelate desuccinylase and cysteinyl-tRNA synthetase	MALDI-TOF/TOF was used to identify proteins implicated in immunological response against mastitis	[144]
Lipids at m/z=1796.2, 1812.2, 1876.2, and 2034.2 Da.	Negative ion MALDI-TOF for rapid diagnosis of colistin resistance termed MALDIxin	[145–147]
Nearly 400 proteins	Proteomic profile of urine samples in dogs with babesiosis	[148]
Approx. 50 proteins differentially expressed	Effect of inulin extract and chicory root on protein expression in the renal cortex and medulla of male pigs	[149]
Fatty acids	Saturated fatty acids are greatest in winter months, and monounsaturated, polyunsaturated, and odd-chain species were present at higher levels in summer	[150]
Ceruloplasmin, serotransferrin, and albumin	Biomarkers of equine conceptus	[151]
Various	MALDI-TOF for genotyping scrapie	[152]
Albumin, clusterin, spermadhesin 1, platelet-activating factor acetylhydrolase, C-C motif chemokine 2 precursor, epididymal-specific lipocalin-5, peptidyl-prolyl cis-trans isomerase, and seminal ribonuclease	Sperm quality and motility with proteomic patterns for Limousin bulls	[153]
42 lipids associated with cell membranes	Effect of diet supplementation with polyunsaturated fatty acids on dairy cow embryo development	[154]

MALDI-TOF=Matrix-assisted laser desorption ionization-time of flight, *E. necatrix*=*Eimeria necatrix*

Ploypetch *et al*. [139] presented a refreshing use of MALDI-TOF. These authors noted that diagnosis of oral melanoma in dogs can be very difficult in early stages as owners may not notice perceptible changes. However, a protein biosignature may exist in the canine’s saliva, which, if detected, may be used for pre-clinical diagnosis. In this work, the authors used MALDI-TOF to identify salivary biomarkers of protein expression present in canine melanoma cases. Follow-up experiments in liquid chromatography–MS allowed further characterization of the protein biomarkers. Yimpring *et al*. [140] used MALDI-TOF to study differential protein expression between pigs with descended versus undescended testes. Interestingly, the authors discovered differential protein expression in tumor necrosis factor receptor superfamily member 18. This protein mediates/induces apoptosis in cells, and identification of this key biomarker suggests a possible role in reduction of fertility and increased risk of testicular malignancies.

Qua *et al*. [141] used MALDI-TOF/MS analysis and MASCOT database searching to identify 50 unique proteins from *Eimeria necatrix* after separation by gel electrophoresis. This organism is of particular importance as it can reduce growth rate in poultry and limit production at harvest. This work did not aim to identify the organism by MALDI-TOF, but rather identify actual proteins within the sample through MS/MS. Wang *et al*. [142] used MALDI-TOF in MS/MS mode to discover three new immunogenic proteins which were protective against porcine *Pasteurella multocida*. Isolated proteins were separated by 2D electrophoresis and exhibited masses between 15 kDa and 100 kDa. Time-of-flight/TOF measurements were pursued with fragments searched against the MASCOT database (www.matrixscience.com) for identification. The article illustrates how MALDI can be used in veterinary research beyond biotyping/identification of microorganisms.

A Colombian team of Sánchez-Jiménez *et al*. [143] used the high-molecular-weight analysis feature of MALDI-TOF to measure the molecular weight and help identify key proteins expressed during an immune reaction to *B. canis*. This ultimately allowed the team to produce recombinant protein in *E. coli* as an effort to develop a sensitive and specific enzyme-linked immunosorbent assay test for *Brucella* infection in humans. In the work of Cunha *et al*. [144], protein signatures from cows with a mastitis history and those vaccinated were considered. Matrix-assisted laser desorption ionization-time-of-flight/TOF was used to identify proteins implicated in immunological response against mastitis. Fifty-nine proteins were identified in the gel electrophoresis experiments and adenosine triphosphate synthase subunit a, succinyl-diaminopimelate desuccinylase, and cysteinyl-tRNA synthetase were found to be potential candidate proteins for the prevention of *S. aureus* mastitis.

**Figure-5:**
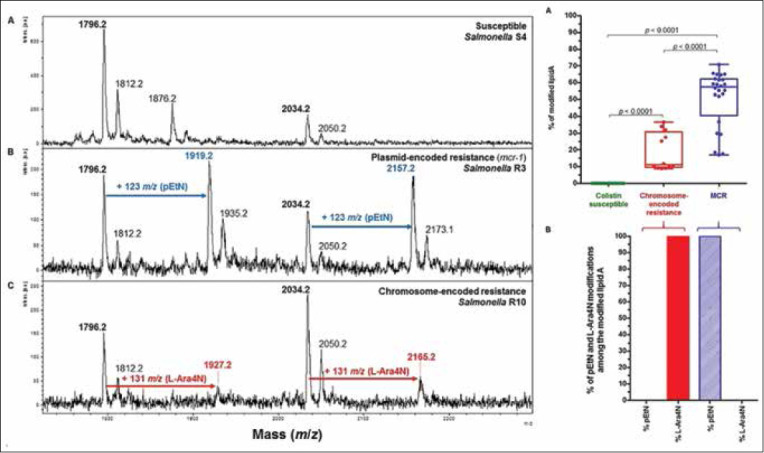
Left – Mass spectra of susceptible and modified *Salmonella enterica* lipid A. (a) Susceptible *S. enterica* lipid A is detected as two peaks; m/z 1796.2 and m/z 2034.2. (b) Lipid A from *S. enterica* exhibiting MCR-medited resistance to colistin exhibit additional peaks at m/z 1919.2 and m/z 2157.2 highlighted in blue. (c) Lipid A from colistin-resistant *S. enterica* isolates carrying chromosomal mutations is modified and detected as additional peaks at m/z 1927.2 and m/z 2165.22 highlighted in red. The presence of these peaks signal antibiotic resistance. Right – (a) Percentage of modified lipid A for colistin-susceptible and colistin-resistant *S. enterica* isolates. Percentage of modified lipid A is represented for colistin-susceptible strains, colistin chromosome-encoded resistant *S. enterica* isolates, and MCR-producing *S. enterica* isolates. (b) Percentage of L-Ara4N and pETN modified lipid A among the global modified lipid A for colistin-resistant *S. enterica* isolates. Figures reproduced from Dortet *et al*. [145] under the terms of the Creative Commons Attribution License (CC BY).

While the vast majority of MALDI measurements occur in the positive ion mode, Dortet *et al*. [145–147] reported using negative ion MALDI-TOF for a rapid diagnosis of colistin resistance termed MALDIxin. In this technique, a small quantity of bacteria is suspended in water and acid hydrolysis is carried out by addition of 2% acetic acid and incubation at 98°C. A small volume of this fluid was directly mixed on the MALDI plate with matrix and dried before analysis to study the lipid profile of the sample. The authors find that *Salmonella enterica* which is susceptible to colistin has a mass spectrum with peaks corresponding to bis-phosphorylated hexa-acyl lipid A, tri-phosphorylated hexa-acyl lipid A and bis-phosphorylated hepta-acyl lipid A present at m/z = 1796.2, 1812.21876.2, and 2034.2 Da. Figure-5 illustrates this result [143]. In colistin-resistant strains, additional peaks were observed in the mass spectrum due to addition of either one phosphoethanolamine group at phosphate position 1 or 4-amino-L-arabinose to the lipid. By tracking the present of lipids chemically modified, a diagnostic test of high confidence is described.

Babesiosis is an infection of red blood cells caused by a parasite which is spread by ticks. The condition often causes kidney injury and Winiarczyk *et al*. [148] studied the proteomic profile of urine samples expressed in dogs with babesiosis. For the urine samples collected from dogs with the condition, MALDI-TOF analysis was used to determine that 373 of 403 proteins were associated exclusively with dogs with babesiosis. The study provides a wealth of information on potential biomarkers of babesiosis in dogs. Feed additives for beef cattle and swine operations are continuously evaluated for affecting the operation’s bottom line. Robak *et al*. [149] have studied the effect of inulin extract and chicory root on protein expression in the renal cortex and medulla of male pigs. Matrix-assisted laser desorption ionization-time-of-flight was used for protein identification. The chicory extract affected expression of 20 renal cortical proteins, while the chicory root affected 44 proteins in the renal cortex. Differentially expressed proteins generally were associated with cellular stress response, heat shock proteins, and carbohydrate metabolism.

Beyond biotyping, ease of sample preparation, and low per-sample cost means MALDI-TOF measurements hold significant promise for chemical analysis related to veterinary science. A Dutch group collected weekly milk samples from 16 dairies to study the seasonal changes in fatty acid composition of milk [150]. The MALDI method allowed characterization of even and odd carbon number and double bond equivalents in triacylglycerides to be determined in collected milk fat. The authors determined that saturated species are greatest in winter months, and monounsaturated, polyunsaturated, and odd-chain species were present at higher levels in summer.

The study of protein expression related to animal reproduction is also an active area of research using MALDI-TOF. Lancheros-Buitrago *et al*. [151] used MALDI-TOF to measure abundance of proteins present in inseminated and cyclic mares. The goal was to identify biomarkers of the potential equine conceptus. Three proteins were identified: Ceruloplasmin, serotransferrin, and albumin (ALB) – which are related to iron regulation and immune response. The work represents a unique use of MALDI for veterinary medicine, in which biomarkers were discovered, given MALDI’s ability to resolve high mass compounds and ionize without fragmentation. In the EU, an active breeding program to increase the frequency of a certain *ARR* allele in sheep populations has been undertaken to control scrapie in sheep (this allele is believed to increase resistance to scrapie). To screen sheep for breeding, a high throughput assay is needed for detection of the allele. While several approaches exist for screening, Diatech Pharmacogenetics developed a product called the Myriapod scrapie kit. This high-throughput assay is based on MALDI-TOF for genotyping at codons 136, 154, 171, 141, and 222 of the small ruminant *PRNP* gene. Migliore *et al*. [152] provided a manuscript which validates the screening kit method for testing of over 12,000 animals in Italy and Sicily. The authors found that 43.9% of sheep had the protective allele, while 12.3% of goats was resistant.

Westfalewicz *et al*. [153] were interested in correlating sperm quality and motility with proteomic patterns for Limousin bulls. In their manuscript, they evaluated seasonal differences in cryopreserved bull semen and found that quality was increased in the autumn and winter season compared to spring and summer. In addition, MALDI-TOF/TOF MS analysis was carried out using a MALDI-TOF tandem mass spectrometer on differentially expressed protein digests excised from a 2D gel. Collected MS/MS spectra of select ions were matched against the MASCOT online server for protein identification. It was found that ALB, clusterin, spermadhesin 1, and a platelet-activating factor acetylhydrolase precursor were most abundant during winter months. C-C motif chemokine 2 precursor, epididymal-specific lipocalin-5, peptidyl-prolyl cis-trans isomerase, and seminal ribonuclease were most abundant during summer, and these proteins may be associated with poor sperm quality. Freret *et al*. [154] wished to study whether diet supplementation with polyunsaturated fatty acids (PUFAs) may affect dairy cow embryo development *in vitro*. The authors fed a supplemented diet before retrieving oocytes from the animals. Then, MALDI-TOF was used on single oocytes and lipid profile was determined. As a conclusion, the authors found that 42 lipids were affected by the diet supplement, with lipids being mainly involved in cell membrane structure. Polyunsaturated fatty acid supplements did enhance oocyte quality, as judged by the authors and modified their lipid composition.

## Matrix-assisted Laser Desorption Ionization-time-of-flight for Chemical Imaging

One of the more exciting emerging areas in which MALDI-TOF is used is chemical imaging. In this experiment, a thin section of tissue is prepared upon a conductive microscope slide. Matrix-assisted laser desorption ionization matrix is then sprayed upon the tissue section, and the sample is dried before being placed within the mass spectrometer. Then, the MALDI laser is rastered across the sample’s surface and mass spectra are recorded at each x and y coordinate. After acquisition of data, a 2D image plot can be recreated from data which illustrates the signal intensity observed at a particular m/z for each location. This allows creation of a 2D image which describes the abundance of a particular substance within the tissue sample. Applications of this technique range from drug distribution studies to the detection of biomarkers in abnormal tissue. Table-4 and the paragraphs below summarize recent MALDI-TOF applications of chemical imaging or mapping presented in the literature which are related to veterinary science [155–159].

**Table-4:** List of recent MALDI-TOF imaging applications related to veterinary science.

Molecules of interest	Notes	References
Formin-like protein (m/z = 1772), eukaryotic translation factor 4H (m/z = 3462), metalloendopeptidase (m/z = 7018), and histone H4 (m/z = 11325)	Bovine follicles and ovary sections were mapped	[155]
Peak at m/z = 329.20 Da (malachite green)	Mapping malachite green (fungicide) in zebrafish tissue	[156]
Moxidectin	Bioaccumulates mainly in the head and eyes of zebrafish embryos at levels up to four orders of magnitude greater than surroundings	[157]
On-tissue protein digest	Trained a model to detect normal versus diseased feline duodenal tissue	[158]
Quinoline antibiotics	Localizing quinolone antibiotics in fish tissue	[159]

MALDI-TOF=Matrix-assisted laser desorption ionization-time of flight

**Figure-6:**
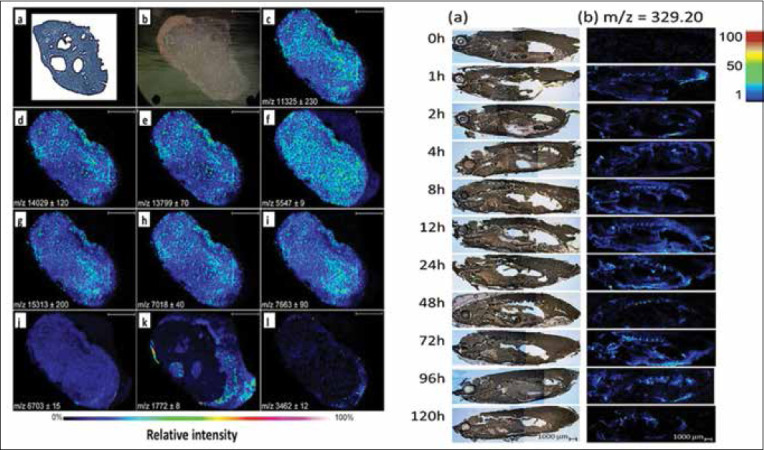
Left – Images of ovarian tissue sectioned in slices (20 μm thick) and placed on glass slides previously covered with gold, coated with a layer of α-cyano-4-hydroxycinnamic acid (α-CHCA) matrix and analyzed on a matrix-assisted laser desorption ionization time-of-flight (TOF)/TOF in positive linear ionization mode. Image maps represent relative abundance of ions of indicated m/z. Reproduced from Westfalewicz *et al*. [153] with permission. Right – Imaging analysis of fish sections with the increased exposure time. (a) The optical images of zebrafish tissue sections, (b) corresponding mass spectrometry images of malachite green (m/z 329.20 tracer) under different sampling times. Reproduced from Freret *et al*. [154] with permission.

The work of Paulini *et al*. [155] highlights an innovative and largely unexplored application of MALDI-TOF measurements in veterinary science – MALDI imaging. In this experiment, the sample is placed on a microscope slide coated with a thin layer of metal and the MALDI laser beam is rastered over the sample while collecting data. The mass spectra collected at specific spatial points can be collated and reconstructed into a 2D image map (Figure-6) [153, 154]. Such an experiment enables chemical histology because the analyst can develop an understanding of the spatial distribution of biomolecules without the need for antibodies or labeling. In Paulini *et al*.’s work, bovine follicles and tissue sections from ovaries were the sample of interest. The authors created image maps for the distribution of several abundant proteins present within the tissue. Based on peaks observed and a literature search, the authors speculated that peaks could correspond to formin-like protein (m/z = 1772), eukaryotic translation factor 4H (m/z = 3462), metalloendopeptidase (m/z = 7018), and histone H4 (m/z = 11325). However, it appears that MS/MS experiments and subsequent MASCOT database searching were not carried out for complete confirmation.

He *et al*. [156] prepared cryosections of 30 μm thick sections of zebrafish for MALDI imaging experiments in which the distribution of malachite green was determined. Malachite green is used as a fungicide in aquaculture. As observed in the right pane of Figure-6, the authors monitored a peak at m/z = 329.20 Da (malachite green tracer) within tissue sections during a 5-day exposure period. The work demonstrates the potential for MALDI-TOF to be used to provide distributions of molecules within animal tissues. This application of MALDI has tremendous potential for veterinary science research and remains largely unexplored.

In a different application of MALDI-TOF, Muniz *et al*. [157] studied the bioaccumulation of a common veterinary antiparasitic drug, moxidectin. The drug affected hatching rates of zebrafish embryos at concentrations in the low microgram per liter range. Matrix-assisted laser desorption ionization imaging was used to determine that moxidectin bioaccumulates mainly in the head and eyes of the embryos at levels up to four orders of magnitude greater than surroundings. The work demonstrates the need for improved control of secondary release of this drug into natural waters.

Marsilio *et al*. [158] reported on use of MALDI-TOF for histology-guided MS. The approach combines the trained eye of board-certified pathologists with the chemical mapping capability of MALDI-TOF. In this demonstration, two 5 mm, feline duodenal tissue sections were cut from biopsy blocks. One section per sample was collected onto an indium-tin oxide-coated glass slide compatible with the mass spectrometer, and the adjacent section was mounted onto a conventional glass slide and areas affected by lymphocytic-plasmacytic enteropathy were annotated by a veterinary pathologist. Porcine trypsin was then sprayed onto the tissue section for on-tissue digestion of proteins, followed by matrix application (after 4 h), and suspect sections of tissue were interrogated by MALDI-TOF. By acquiring data for normal versus affected areas, the authors were able to train a linear discriminate machine learning model to detect normal versus affected tissues. The resultant model exhibited a sensitivity, specificity, and accuracy of 86.7%, 91.7%, and 88.9%. This work outlines an application of MALDI-TOF which is truly in its infancy of development.

Braga *et al*. [159] reported that MALDI-TOF can be used to measure a variety of quinolone antibiotics in fish tissue. This work is out of the norm since MALDI is usually thought to be subject to low-molecular-weight interferences due to matrix peaks. In addition, MALDI often struggles to produce reproducible signal intensities and can be subject to matrix effects. The authors comment that no low mass interference was observed for the quinolones tested, andthe matrix effect was alleviated through the use of an internal standard (enrofloxacin-d) which ionizes similarly to the analytes.

In addition to the works cited above, tissue imaging by MALDI-TOF is an actively growing area of research. The field has been discussed/reviewed several times in the previous literature [160–162]. Matrix-assisted laser desorption ionization imaging coupled with guided histology presents exciting opportunities for veterinary research.

## Discussion

Clearly, over the past decade, MALDI-TOF has emerged as a key veterinary diagnostic tool. However, this manuscript would be remiss without considering both current strengths and limitations of the technique. To begin this discussion, consider Table-5, which is a list of common veterinary diagnosis for which MALDI-TOF may be applied [3, 20, 89, 163–205]. This list of diagnosis was generated by considering the content knowledge required for the North American Veterinary Licensing Examination. Within this framework of discussion, only 44% of crucial diagnoses are currently tractable using MALDI-TOF as a diagnostic test. This statistic even ignores specificity/sensitivity issues related to the diagnostic test itself. We can see that considerable progress has been made in employing MALDI-TOF for diagnosis, particularly in the realm of bacterial infection. However, the MALDI-TOF diagnostic technology lags far behind for viral and parasitic infections. This gap in implementation is logical; viruses are generally not included within commercially available search databases and may not present easily distinguishable patterns of proteins required for identification. On the other hand, parasite samples generally offer the analyst an abundance of proteins for identification; however, tissues are often quite recalcitrant and resistant to digestion/homogenization. More extensive sample preparation steps are required for parasite identification, and in addition, the development of parasite spectral reference libraries is in its infancy.

**Table-5:** Common veterinary diagnosis and development state of MALDI-TOF for detection.

Condition	MALDI-TOF diagnostic	Example reference
*Aeromonas hydrophila*	Yes	[163]
African horse sickness	Knowledge gap	
Aleutian disease	Gap/needs development	[164]
Anaplasmosis	Knowledge gap	
Anthrax	Yes	[165]
Arbovirus	Knowledge gap	
Argulus	Knowledge gap	
*Ascaris suum* infection	Yes	[166]
Aspergillosis	Yes	[167]
Babesiosis	Knowledge gap	
Blastomycosis	Knowledge gap	
Blue tongue virus	Knowledge gap	
*Bordetella bronchiseptica*	Yes	[168]
Botulism	Yes	[89]
Bovine respiratory disease	Yes	[169]
Bovine viral diarrhea virus	Knowledge gap	
Brucellosis	Yes	[170]
Candidiasis	Yes	[171]
Chlamydiosis	Knowledge gap	
Chronic wasting disease	Knowledge gap	
Circovirus	Knowledge gap	
Clostridiosis	Yes	[172]
Coccidiosis	Knowledge gap	
Columnaris	Yes	[173]
Consumption of moldy hay	Knowledge gap	
Coronavirus	Yes	[174]
*Corynebacterium renale*	Knowledge gap	
Cryptococcosis	Yes	[175]
Distemper	Knowledge gap	
Endocrine conditions	Knowledge gap	
Epizootic catarrhal enteritis	Knowledge gap	
Equine encephalomyelitis	Knowledge gap	
Equine piroplasmosis	Knowledge gap	
*Escherichia coli*	Yes	[176]
Favus	Knowledge gap	
Feline immunodeficiency virus	Knowledge gap	
Feline infectious anemia	Knowledge gap	
*Feline leukemia*	Knowledge gap	
*Feline panleukopenia*	Knowledge gap	
Foot and mouth disease	Knowledge gap	
Fowlpox	Knowledge gap	
*Giardia*	Yes	[177]
Glanders	Yes	[178]
Heartworm	Knowledge gap	
Heat stroke	Knowledge gap	
Helminths and flukes	Yes	[3]
Hepatitis	Knowledge gap	
Histoplasmosis	Yes	[179]
Influenza	Knowledge gap	
*Lernaea*	Knowledge gap	
*Lawsonia* spp. infection	Knowledge gap	
Leptospirosis	Yes	[180][181][182]
Leukosis	Knowledge gap	
Listeriosis	Yes	[183]
Marek disease	Knowledge gap	
Mastitis	Yes	[184]
Methicillin-resistant *Staphylococcus aureus*	Gap/under development	[185]
Monkeypox	Knowledge gap	
*Monogenea*	Progressing/in principle	[186]
*Moraxella bovis*	Yes	[187]
Mycobacteriosis	Yes	[188]
*Mycoplasma hyopneumoniae*	Knowledge gap	
Newcastle disease	Yes	[189]
Pacheco disease	Knowledge gap	
Pacheco’s Disease	Knowledge gap	
Paratuberculosis	Yes	[190]
Parvovirus	Knowledge gap	
*Pasteurella multocida*	Yes	[191]
Pigeon fever	Knowledge gap	
Polioencephalomalacia	Knowledge gap	
Polyomavirus	Knowledge gap	
Potomac horse fever	Knowledge gap	
Psittacosis	Knowledge gap	
Pyelonephritis	Yes	[192][193]
Pyometra	Knowledge gap	
Rabies	Yes	[194]
Rhinitis	Knowledge gap	
*Rhodococcus equi*	Yes	[195]
Ringworm	Knowledge gap	
*Rotavirus*	Knowledge gap	
Rubella	Knowledge gap	
Salmonellosis	Yes	[196]
Scrapie	Knowledge gap	
Seneca valley virus	Knowledge gap	
Sepsis	Under development	[197]
Strangles	Yes	[198]
Streptococcosis	Yes	[199]
*Streptococcus suis* infection	Yes	[200]
Swine fever	Knowledge gap	
Tetanus	Knowledge gap	
Toxoplasmosis	Knowledge gap	
Trichinosis	Yes	[201]
Trichomoniasis	Yes	[202]
Tritrichomoniasis	Knowledge gap	
Trypanosomiasis	Yes	[203]
Tuberculosis	Yes	[204]
Tularemia	Yes	[205]
Tyzzer disease	Knowledge gap	
Vibriosis	Yes	[20]
West Nile encephalomyelitis	Knowledge gap	

MALDI-TOF=Matrix-assisted laser desorption ionization-time of flight.

The main advantage of MALDI-TOF for diagnosis is the low-cost per sample. For a developed method conducted at a high-throughput diagnostic center, cost per sample can be under $1 per sample. The method itself is also very tolerant to a wide variety of sample matrices, requires very small amounts of sample (under 1 μL), and minimal sample preparation steps are required. As indicated above, typically sample is mixed with a matrix solution on a stainless-steel plate and dried before analysis. Very few other analytical methods feature such minimal sample preparation. Because of the technique’s simplicity, the measurement itself can be conducted very quickly by personnel with only moderate training and offers high-throughput analysis with rapid turnaround times. Of course, a limit to this premise is the time required for culturing bacteria, which is often needed before analysis.

At present, sequencing the 16S rRNA gene sequence is the gold standard utilized for pathogen detection and microecological research. The 16S technique has been reported to obtain as high as 100% sensitivity and specificity for determining *Pseudomonas* species [206]. In addition to the high sensitivity and specificity demonstrated, the ability to amplify sequences through the polymerase chain reaction helps assure a low copy number of genetic material may be detected. While 16S sequencing may allow for excellent sensitivity and specificity metrics, the cost per sample is roughly $20–100, much higher than what is possible with MALDI-TOF. In addition, sample turnaround time can be far shorter using MALDI-TOF. In general, MALDI-TOF technology is preferred when rapid “quick look” at a specimen is needed and 16S sequencing and MALDI-TOF may be used in concert for unequivocal confirmation of diagnosis. In addition to providing veterinarians with rapid results to guide care, MALDI-TOF would also be a great option for screening of livestock or companion animals crossing political boundaries [207].

Despite the advances in MALDI-TOF, some caveats remain. In the arena of species identification, individual research groups are developing spectral libraries for their own applications. To broaden the applicability of MALDI-TOF, a mechanism for a combination of existing knowledge – vis-à-vis spectral libraries should be pursued. There must be a means to pool investigator developed spectral libraries across boundaries of geography, instrument platform, and instrument manufacturer. No one research group can collect all spectral libraries possible and unleashing the true diagnostic power of MALDI-TOF requires dissemination of spectral libraries to the broader research community. This is the major current limitation of MALDI-TOF for microorganism identification. Collaborative, a multinational effort is now required to broaden the impact of MALDI-TOF technology by developing and disseminating mass spectral libraries. Achieving this goal should be coordinated and pursued vigorously by science funding agencies.

For the general theme of MALDI-TOF for entomological identification, sample preparation routines are a major issue affecting research. How should insects be dissected, and which body parts should be used for analysis? Can insects be stored in solvents such as ethanol before MALDI-TOF? What solvent combinations and concentration of acid are necessary to extract identifiable proteins from the samples? How should the dissected body parts be ground up or blended within the solvent? Many papers have established that insects can be identified by MALDI-TOF; however, identification may be dependent on specific protocols. Developing consistent sample preparation protocols among investigators would allow better matching of databases between laboratories.

An area of opportunity for MALDI-TOF’s growth in the veterinary sciences is the application of machine learning algorithms to the MALDI-TOF data stream. Machine learning approaches to classify, cluster, or predict outcomes based on data can advance molecular-based diagnostics and help identify novel biomarkers indicative of diseased states. However, it is not typical or common that an individual will simultaneously possess training in a scientific discipline such as biology or chemistry and have adequate knowledge of machine learning to implement sophisticated models. This field can be advanced by providing MALDI-TOF users access to training in software to achieve the machine learning model.

Finally, a tremendous amount of opportunity exists in using the chemical nature of the MALDI-TOF data stream more. Remarkably, for diagnostics, the chemical information provided by the mass spectrum is essentially disregarded and only pattern recognition is used to identify an organism. For biotyping, the identity of the protein itself and its mass is typically not important to the analyst. This essentially discards high-quality biochemical information related to the sample under study. It is likely considerable knowledge can be generated by more careful consideration of the chemical nature of the MALDI-TOF mass spectrum and use of advanced tools such as collisional dissociation or in-source dissociation of biomolecules. Matrix-assisted laser desorption ionization imaging of tissue sections also is an exciting area of research since a trained pathologist’s eye, combined with a light microscope, and MALDI-TOF data stream can uncover novel biomarkers of abnormal tissues. As devices for MALDI imaging become more available, faster, and more user-friendly, I expect this area of research to become more popular. At present, spatial mapping of tissues often is hampered by required original programming of laser raster patterns, long data acquisition times (hours), and manual reconstruction of chemical images.

## Conclusion

Matrix-assisted laser desorption ionization-time-of-flight is impacting the field of veterinary diagnosis. While the identification of bacterial microorganisms remains the largest application area of the technique, exciting new avenues of research are being developed. Table-5 lists remaining challenges for the application of MALDI-TOF for veterinary diagnoses. Given the advantages of a low-cost per sample, extremely simple sample preparation, and ease of instrument operation, the author fully expects the continued development of the technique over the next decade. Surely, further validation of MALDI-TOF-based methods for additional clinical diagnoses will create a versatile tool for veterinary practices.

## Data Availability

The supplementary data can be available from the corresponding author upon reasonable request.

## Authors’ Contributions

JET: Conceived the work, researched the literature, and drafted and revised the manuscript. He has read and approved the final manuscript.
